# Functional genomics of lactic acid bacteria: from food to health

**DOI:** 10.1186/1475-2859-13-S1-S8

**Published:** 2014-08-29

**Authors:** François P Douillard, Willem M de Vos

**Affiliations:** 1 Department of Veterinary Biosciences, University of Helsinki, Agnes Sjöberginkatu 2, 00790 Helsinki, Finland; 2 Laboratory of Microbiology, Wageningen University, Dreijenplein 10, 6703 HB Wageningen, The Netherlands; 3 Department of Bacteriology & Immunology, University of Helsinki, Haartmaninkatu 3, 00014 Helsinki, Finland

**Keywords:** Genome comparison, evolution, adaptation, applied genomics, pili, respiration

## Abstract

Genome analysis using next generation sequencing technologies has revolutionized the characterization of lactic acid bacteria and complete genomes of all major groups are now available. Comparative genomics has provided new insights into the natural and laboratory evolution of lactic acid bacteria and their environmental interactions. Moreover, functional genomics approaches have been used to understand the response of lactic acid bacteria to their environment. The results have been instrumental in understanding the adaptation of lactic acid bacteria in artisanal and industrial food fermentations as well as their interactions with the human host. Collectively, this has led to a detailed analysis of genes involved in colonization, persistence, interaction and signaling towards to the human host and its health. Finally, massive parallel genome re-sequencing has provided new opportunities in applied genomics, specifically in the characterization of novel non-GMO strains that have potential to be used in the food industry. Here, we provide an overview of the state of the art of these functional genomics approaches and their impact in understanding, applying and designing lactic acid bacteria for food and health.

## Introduction & outline

**Figure 1:**
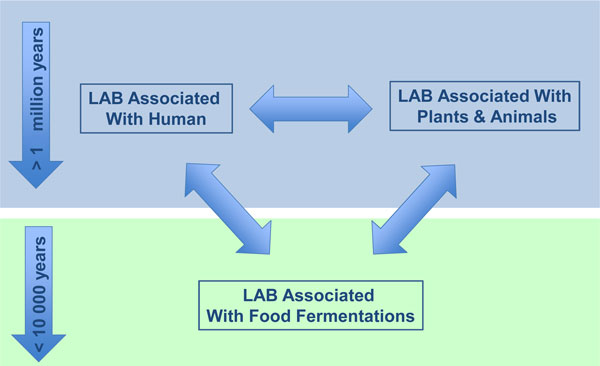
**Overview of LAB associations with plants and animals, human and foods**. The estimated time frames of the evolutionary events relating to the emergence of human (top) and domestication (bottom) are indicated - please note their different dimensions. For a further explanation, see text.

Lactic acid bacteria (LAB) and humans share a long and intricate history. Well known are the first food fermentations reported in ancient times that contributed to the preservation and quality improvement of raw plant, meat and milk substrates. Most likely the transition from hunter-gatherers to an agricultural lifestyle, some 10,000 years ago, contributed to the further development of these food fermentations that are now practiced worldwide on an industrial scale. However, our interactions with LAB are more intimate and have a much longer history than the food fermentations that were initiated by the LAB present at that time (Figure 1). In addition to many plants and animals, the human body is also colonized by LAB and early culturing studies already documented the presence of LAB at different locations, *e.g*. the gastro-intestinal tract or the oral cavity [1]. However, many microbes cannot yet be cultured and this also holds for LAB [2]. Until recently, technological limitations precluded the global characterization of human microbiota in terms of composition, diversity and dynamics. Massive parallel sequencing and other high throughput approaches have offered novel ways to explore and examine the microbiota from different human body cavities [3-5]. Much attention has been given to the human gastro-intestinal (GI) tract but the number of endogenous (autochtonous) LAB in the human system is rather low (Douillard and De Vos, in press; see also below). This contrasts with many animals where the GI-tract is a well-established habitat for high numbers of endogenous LAB, such as the fore-stomach of mice and other rodents, as well as the crop of chicken and other birds [6,7]. Hence, these animal systems, similar to many plants that are colonized with LAB in the phyllosphere, may constitute reservoirs for LAB found in food fermentations or even the human body (Figure 1).

In retrospect, it may be argued that the low level of endogenous LAB in human explains the impact of passenger (allochtonous) LAB on the human host, as exemplified with LAB that are marketed as probiotics and after consumption have shown to provide health benefits [8,9]. The continuing consumer interest in these and other LAB-containing functional foods may be a reason for the special fondness for these bacteria that go beyond any personal affection [10]. This interest has a long history as the first association of LAB with traditional fermentations, naturalness and long life, has been described over 100 years ago for what is now known as *Lactobacillus delbrueckii *subsp. *bulgaricus *[11]. Moreover, it is widely known that LAB are highly versatile and include phylogenetically related bacterial taxa that are essentially non-pathogenic.

**Figure 2:**
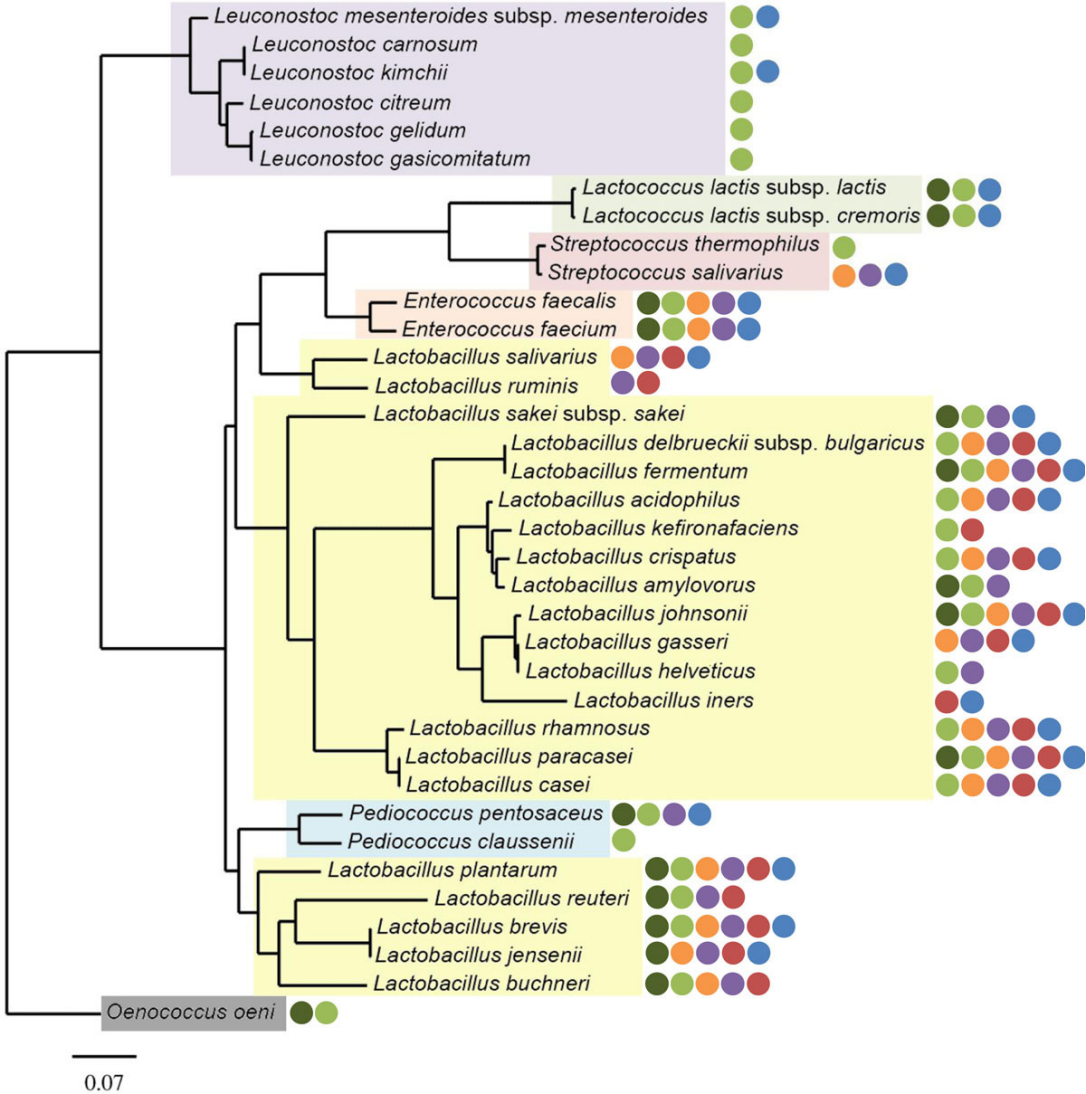
**A phylogenetic tree of based on sequences of 7 housekeeping genes (*rec*A, *rpo*D, *dna*K, *inf*C, *rpl*A, *rps*B and *rpm*A) from the 36 LAB species**. The tree was generated using previously described computational methods [210-219]. Species were colored according to their genus (purple, *Leuconostoc *spp. ; yellow, *Lactobacillus *spp. ; blue, *Pediococcus *spp.; green, *Lactococcus *spp.; pink, *Streptococcus *spp. ; orange, *Enterococcus *spp. ; grey, *Oenococcus *spp. ). In addition, the presence of isolates in a particular niche are indicated by colored dots (dark green, plant material; green, food products; orange, oral cavity; purple, gastro-intestinal tract; magenta, vaginal cavity and blue, other body sites and clinical isolates). This illustrates the ecological versatility of each species but does not further detail its ecological role, *i.e*. transient (allochthonous) or endogenous (autochthonous).

The early days of the genome sequencing era witnessed a strong focus on pathogens, starting with *Haemophilus influenza *in 1995 [12]. In hindsight, this medical focus explains why the first LAB genomes were only deciphered some years later, in the early 2000s with *Lactococcus lactis *subsp. *lactis *[13] and *Lactobacillus plantarum *[14]. Ever since, the number of sequenced LAB genomes has grown exponentially and currently genomic data from over 100 LAB species and strains are available in various public databases. These offer a wealth of information, to further understand LAB with respect to their gene content, their properties, and their ecological role in human health as well as in food fermentations [15]. The present review aims at discussing and describing the latest functional genomic advances in LAB species that are associated with food and health (Figure 1). As prototype functional genomics studies rely on a complete genome sequence, we focus here on the LAB that comply with this criterion and these include rod-shaped LAB (*Lactobacillus*) and a dozen coccoid LAB, including *Lactoccoccus, Streptococcus, Enterococcus, Pediococcus *spp. and *Oenococcus *spp. (Table 1). Remarkably, this morphological distinction is reflected in a dichotomy in the genome-based phylogenetic tree (Figure 2). We will specifically focus on food-related fermentations where much basic progress has been on the global expression control using transcriptome and proteome approaches that are facilitated by the fact that these systems are easily accessible or can be mimicked in the laboratory. In contrast, the human associated LAB are more difficult to access and most studies that will be discussed relate to LAB with clear health benefits to the human host. Finally, we will address the evolutionary impact of the genomic adaptations (Figure 1) and describe some of the latest genomics approaches applied to LAB for improved food fermentations or health benefits.

**Table 1:** Genomic features of a selected number of lactic acid bacteria related to human lifestyle and health.

Bacterial species	Example of Sequenced Strain	Isolation Source	Genome Size (Mbp)	Number of Plasmids	%GC	Number of Proteins	References
***Lactobacilli***							
*Lactobacillus acidophilus*	NCFM	GI tract (Feces)	1.99	0	34.7	1,832	[112]
*Lactobacillus amylovorus*	GRL1112	Pig GI Tract (Feces)*	2.13	2	38.1	2,121	[195]
*Lactobacillus brevis*	ATCC 367	Unknown	2.34	2	46.0	2,218	[19]
*Lactobacillus buchneri*	ATCC 11577	Oral Cavity	2.86	n.d.	39,5	3,002	DS
*Lactobacillus casei*	BL23	Food (Cheese)	3.08	0	46.3	2,997	[196]
*Lactobacillus crispatus*	EM-LC1	GI Tract (Feces)	1.83	n.d.	37.0	1,751	DS
*Lactobacillus delbrueckii *subsp. *bulgaricus*	ATCC 11842	Food (Dairy product)	1.87	0	49.7	1,529	[19]
*Lactobacillus fermentum*	IFO 3956	Food (Plant)	2.1	0	51.5	1,843	[197]
*Lactobacillus gasseri*	ATCC 33323	Human origin	1.89	0	35.3	1,755	[19]
*Lactobacillus helveticus*	DPC 4571	Food (Cheese)	2.08	0	37.1	1,610	[113]
*Lactobacillus iners*	AB-1	Vaginal Cavity	1.29	0	32.7	1,209	[154]
*Lactobacillus jensenii*	269-3	Vaginal Cavity	1.69	n.d.	34.4	1,575	DS
*Lactobacillus johnsonii*	NCC 533	GI Tract (Intestine)	1.99	0	34.6	1,821	[198]
*Lactobacillus kefiranofaciens *subsp. *kefiranofaciens*	ZW3	Food (Kefir)	2.35	2	37.4	2,162	[199]
*Lactobacillus paracasei *subsp. *casei*	N1115	Food (Dairy products)	3.06	4	46.5	2,985	[200]
*Lactobacillus plantarum*	WCFS1	Oral Cavity (Saliva)	3.35	3	44.4	3,063	[201]
*Lactobacillus reuteri*	DSM 20016	GI Tract (Intestine)	2.0	0	38.9	1,900	DS
*Lactobacillus rhamnosus*	GG	GI Tract (Intestine)	3.01	0	46.7	2,913	[69]
*Lactobacillus ruminis*	ATCC 25644	GI Tract (Intestine)	2.07	0	43.7	2,153	[111]
*Lactobacillus salivarius *subsp. *salivarius*	UCC118	GI Tract (Intestine)	2.13	3	33.0	2,013	[106]
*Lactobacillus sakei *subsp. *sakei*	23K	Food (Meat)	1.88	0	41.3	1,871	[202]
***Lactococci***							
*Lactococcus lactis *subsp. *lactis*	IL1403	Food (Cheese)	2.37	0	35.3	2,277	[13]
*Lactococcus lactis *subsp. *cremoris*	MG1363	Food (Dairy Products)	2.53	0	35.7	2.434	[203]
***Streptococci***							
*Streptococcus salivarius*	CCHSS3	Oral Cavity	2.22	0	39.9	2,027	DS
*Streptococcus thermophilus*	CNRZ1066	Food (Yoghurt)	1.8	0	39.1	1,914	[181]
***Enterococci***							
*Enterococcus faecalis*	V583	Clinical Sample (Blood)	3.36	3	37.4	3,264	[204]
*Enterococcus faecium*	DO	Clinical Sample	3.05	3	37.9	3,114	[205]
***Oenococci***							
*Oenococcus oeni*	PSU-1	Food (Plant)	1.78	0	37.9	1,691	[19]
***Pediococcus***							
*Pediococcus pentosaceus*	ATCC 25745	Food (Plant)	1.83	0	37.4	1,752	[19]
*Pediococcus claussenii*	ATCC BAA-344	Food (Beer)	1.98	8	37.0	1,881	[182]
***Leuconostoc***							
*Leuconostoc mesenteroides*	ATCC 8293	Food (Olives)	2.08	1	37.7	2,003	[19]
*Leuconostoc citreum*	KM20	Food (Kimchi)	1.9	4	38.9	1,820	[136]
*Leuconostoc gelidum*	JB7	Food (Kimchi)	1.89	0	36.7	1,796	[206]
*Leuconostoc carnosum*	JB16	Food (Kimchi)	1.77	4	37.1	1,691	[207]
*Leuconostoc kimchi*	IMSNU 11154	Food (Kimchi)	2.1	5	37.9	2,129	[208]
*Leuconostoc gasicomitatum*	LMG 18811T	Food (Spoilage)	1.95	0	36.7	1,912	[209]

Legend: DS, Direct Submission to sequence databases; n.d, not defined; *, No human isolates have been sequenced yet. Due to some discrepancies between the original references and the sequence databases, the data shown in the table were exclusively retrieved from NCBI databases as on 4th of April 2014.

## Functional genomics of LAB in food fermentations

The use of LAB in industrial fermentations represents a multi-billion dollar industry with the dairy products cheese and yoghurt as the most produced commodities [16]. Hence, considerable attention is given to the function of LAB during the fermentation of milk into the final product. The most important LAB used as starters in these dairy fermentations are *Lactococcus lactis, Streptococcus thermophilus, Lactobacillus delbruekii *subsp. *bulgaricus*, while in some cases also some *Leuconostoc *or other *Lactobacillus *spp. are used. Representative strains of these starter bacteria have been genomically characterized (Table 1) [16]. However, in many cases the genome sequences of industrial starter strains have not been determined yet or not been made available in public databases. This is exemplified by the case of the cheese starters that in most cases belong to *Lactococcus lactis *subsp. *cremoris*. In addition to the genomes of strain MG163 and its derivative NZ9000, widely used as a host with the NICE system [17,18], only 4 other complete genomes of this taxon have been reported. These genomes include their plasmid complement, which is of crucial importance as it harbors many important dairy functions [16]. The first strain was SK11, a well-studied good flavor-producing strain used as model in earlier genetic studies [19,20]. More recent examples include strain A76, isolated from a cheese production system and strain UC 509.9, an Irish starter with the smallest genome [21]. Moreover, the complete genome of *Lactococcus lactis *subsp. *cremoris *KW2, derived from a corn-fermentation, has been elucidated [22]. This and another plant isolate, *Lactococcus lactis *subsp. *lactis *KF147, isolated from mung bean sprouts, with one of the largest lactococcal genomes [23], serve as models for domestication studies (Figure 1) and will be discussed below.

In recent years genomic interest has developed into the so called Non-Starter LAB (NSLAB) that are naturally present in dairy fermentations and in some cases have been developed into adjunct starters that contribute to flavor development or quality improvement of fermented foods [24,25]. An example is the recent genomic characterization of *Lactobacillus helveticus *strain CNRZ 32, used as an adjunct starter to reduce bitterness and found to encode 4 different cell-envelope proteinases, in contrast to other Lactobacilli that have one or none [26].

A variety of functional genomics approaches have been reported in the last decade that relate to LAB found in food fermentations. Most have focused on the dairy LAB and here we will discuss the salient features of the common elements that relate to the control of gene expression and serve as models for other LABs. Moreover, functional studies have targeted a variety of foods where attention has been focused on starter LAB, NSLAB and spoilage LAB. Finally, in these studies a series of discoveries have been described that affect the lifestyle of LAB and these are briefly summarized.

### Growth & global regulation

LAB are known to be rather fastidious bacteria that compete based on rapid growth and lactic acid production in a selected number of habitats (see Figure 2) Genomic-based metabolic reconstructions and modeling have confirmed the dependence on external sources of sugar and protein that are found in complex media such as milk, meat and some plant materials. So much attention has been focusing on the control of carbon and nitrogen metabolism.

By far the most important factor controlling sugar degradation in LAB is the catabolite control protein CcpA. The first *ccpA *gene of LAB was discovered in *Lactococcus lactis *MG1363 and found to act as a transcriptional activator of the lactic acid synthesis (*las*) operon with the order *pfk-pyk-ldh *[27]. Using sensitive microarray analysis in wild-type MG1363 and an isogenic *ccpA *deletion strain, the time-dependent global regulon was uncovered and allowed the identification of 82 CcpA binding sites, known as catabolite responsive elements (*cre)*, predicting the role of CcpA in sugar transport and other metabolic processes [28]. Recently, a high-resolution crystal structure of the 76 kDa homodimer has been solved and a first analysis of the interaction between the *cre *sites and CcpA has been made for the cellobiose operon [29,30]. New aspects on the role of CcpA in global control are continuously being uncovered by using transcriptional and proteomic studies in many LAB [31-35]. Moreover in other cocci besides *Lactoccocus *spp., CcpA is an important control system, as demonstrated in *Streptococcus thermophilus *and *Enterococcus faecalis *[36,37]. In an elegant metabolic and transcriptional study it was recently found that resting cells of MG1363 at pH 5.1 showed enormous pools of lactic acid, reaching levels of 700 mM inside the cells [38]. Apart from various stress-response genes and the membrane bound ATPase genes, also various glycolytic genes belonging to the *las *operon were overexpressed. Another recent study addressed the transcriptional network of *Lactococcus lactis *MG1363 in milk and identified CcpA as one of the major regulators in addition to others that are discussed below. Moreover, 2 new potential CcpA target sites were identified and are suggested to be involved in fine tuning of the CcpA mediated control [39]. The organization of the *ccpA *gene in many LAB is such that it is juxtaposed but divergently transcribed from the prolidase-encoding *pepQ *gene, indicating a link between carbon and nitrogen metabolism, as first observed in *Lactococcus lactis *MG1363 [28]. While carbon control is highly relevant for LAB, the tight control of nitrogen metabolism may be even more important as amino acid synthesis is a costly cellular process.

Several nitrogen control systems are present in LAB and the most studied include GlnR and CodY. While GlnR is present in all LAB genomes, CodY is only present in *Lactocccus, Streptococcus *and *Enterococcus *spp. [40]. A comparative genomic study of GlnR regulon, revealed its target site to be present in all LAB genomes and, supported by published transcriptome analyses, predicted GlnR to be involved in controlling the import of nitrogen-containing compounds and the synthesis of intracellular ammonia under conditions of high nitrogen availability [40]. In *Lactococcus lactis *MG1363 GlnR was found to be rather specific but CodY appeared to be a much more global control system [41]. This appeared to be the cases for all other coccoid LAB where it is present. Similar to the identification of the CcpA regulon, a comparative transcriptome approach using an isogenic *codY *mutant was followed to identify the CodY regulon in *Lactococcus lactis *MG1363 [42]. Over 30 genes mainly involved in amino acid metabolism were identified to be under control of CodY in strain MG1363 and in later study in strain IL1403 some more were predicted based on the CodY target (CodY box) in the genome of this strain [43]. The CodY box is present in the promoter of the *codY *gene, explaining that *codY *regulates its own synthesis and does so in response to branched chain amino acids [42]. Importantly, CodY controls the proteolytic system of *Lactococcus lactis *and notably the cell-wall proteinase (PrtP), the key enzyme in milk degradation that prior to the genomic era was shown to be controlled at the transcriptional level by milk-derived peptides [44]. During growth of strain MG1363 in milk, CodY also acts as a regulator of a major network and detailed transcriptional studied identified a second CodY box in the intergenic regions of 3 operons but the function of this element remains enigmatic in absence of further experimental work [39]. An integrated transcriptomic and proteomic analysis of the adaptation of strain IL1403 to isoleucine starvation showed that CodY was specifically dedicated to the control of the supply of this branched chain amino acid [45]. In *Streptococcus thermophilus *CodY was found to be also involved in the control of the proteolytic system but the study failed to identify a conserved CodY box, indicative of a species-specific *cis-*acting control elements [46]. Remarkably, CodY in pathogenic Streptococci was shown to provide a link between amino acid and carbon metabolism as well as virulence factors such as nasopharynx colonization and the synthesis of exoproteins [47,48]. It would be of interest to determine whether CodY of *Streptococcus salivarius *has a similar role in the colonization of this and related species in the oral or other human related cavities (see below). The absence of a *codY *gene in the genomes of *Oenococcus *and *Pediococcus *suggests that these bacteria have a life style where they do not need such an intricate protein control [40]. Alternatively, these bacterial species may employ different regulatory mechanisms, possibly involving unrecognized regulators.

Apart from the above-mentioned CcpA, GlnR and CodY, many other specific and global regulators have been described and functionally studied. In many cases new links may be observed as the control systems all seem to be interlinked. With the development of high throughput transcriptome and RNAseq approaches, new avenues to identify and map these are emerging. The recent analysis of the global regulatory networks, identified during growth of *Lactococcus lactis *subsp. *cremoris *MG1363 in milk, is such an example [39]. This is expected to be followed by other studies that will provide insights into the global control, the *cis-*acting elements, and their nodes. The challenge is to relate these transcriptional networks to the metabolic networks that are now well-developed to increase the predictability of LAB in the model systems, in food products and in association with human [49].

### Expression in foods

To improve the understanding of growth and function of LAB in fermented foods, numerous global transcriptional, proteomic and recently also metabolomic studies have been performed. Model and starter strains of *Lactococcus lactis *have been the first to be studied. A lactose-proficient derivative of the model strain MG1363 was used in an artificial cheese system using an expression technology approach [50]. While a series of genes involved in amino acid transport and metabolism were identified, the approach suffered from the fact that the strain used was plasmid-free and did not contain the PrtP-encoded system and hence was not proteolytic. This caveat also applies to the elegant study of strain MG1363 in milk elucidating the global networks [39]. However, several other studies have addressed the expression in cheese of starter lactococci that are capable of rapid growth in milk. Using cheeses made from milk concentrated by ultrafiltration (UF-Cheese) and the starter *Lactococcus lactis *subsp. *lactis *biovar diacetylactis LD6, a detailed study was made of the *in situ *global gene expression [51]. Genes of the proteolytic system were increased due to down-regulation of CodY repression, while acid and oxidative stress-related genes were increased. Moreover, carbon limitation was apparent and involved release of CcpA-mediated control. In similar UF-Cheeses made with strain LD6, recently the metabolites were determined using an unsupervised mass-spectometry approach, illustrating the power of other non-targeted functional approaches [52]. In an unrelated study, four *Lactococcus lactis *subsp. *cremoris *starter strains (SK11, and proteolytic variants of HP, Wg2 and E8) were used in parallel cheese vats and analyzed for their transcriptomic response [53]. This resulted in the definition of a core transcriptome with almost 200 genes, mainly encoding for house-keeping functions but also those involved in cysteine metabolism. Several of these were found to be under control of the CodY regulator, reiterating the common theme discussed above. As indicated below, correlations between CcpA, CodY and the stringent response exist and it is expected that these regulatory circuits are all operating during these complex fermentations in cheese making. As often mixtures of LAB strains are used as cheese starter cultures, various approaches have been developed to differentiate between the components of the starter. Various metagenomics and quantitative PCR approaches have been tested and shown to have potential for strain differentiation or expression [54,55]. Sequence analysis of 16S rRNA transcripts has recently been used to identify the microbial composition and activity of Cheddar cheese batches, identifying both LAB and NSLAB. These and similar investigations can be coupled to RNAseq studies to analyze the expression in real time of the different components.

Only few other global gene expression studies have been performed in food products other than those derived from fermented milk. The global transcriptome of *Lactococcus garviae*, a fish and opportunistic human pathogen was analyzed and revealed a heme-dependent and cold-induced respiration system [56]. This had already been described some years ago in another strain of *Lactococcus garviae *[57]. Such a respiration system was also identified in a transcriptomic approach of *Leuconostoc gasicomitatum*, an emerging food spoilage organism, when grown in meat [58]. The endogeneous heme present in meat allowed respiration and this increased growth rate and yield. Interestingly, this had no impact on the transcriptional response of *Leuconostoc gasicomitatum*, similar to what has been observed in *Lactococcus lactis *[59]. However, it has been described that the meat-grown *Leuconostoc gasicomitatum *respiration activity was increased 1000-fold and was paralleled by the production of different metabolites, suggesting that its control is at the metabolic rather than the transcriptional level [58].

### Novel insight and functions

While providing a molecular understanding of the adaptation of LAB to the food environment, the genomics studies discussed here also present insight in novel functions. An example is the identification of a novel stress regulon under the control of the protein Ldb0677 in *Lactobacillus delbrueckii *subsp. *bulgaricus *by using a proteomic approach and its characterization by molecular techniques [60]. Moreover, studies in other model systems may shed new light on the findings in LAB. One such new insight derives from findings in *Bacillus subtilis*, which reportedly shares a common ancestor with the LAB [19]. It has recently been shown that CcpA forms complexes with CodY in *Bacillus subtilis *and there is no reason to assume this would not be possible in LAB [61]. This strongly suggests that the carbon and nitrogen control in LAB are intimately connected. Similarly, structural analysis of the *Bacillus subtilis *CodY indicated that GTP is a ligand for this conserved regulator and hence CodY reacts to (p)ppGpp levels formed in the stringent response [62-64]. The stringent response of the (p)ppGpp alarmone may well be one of the general triggers that operate in LAB during cheese fermentation.

The discovery of aerobic respiration in LAB and its genetic elucidation has been well documented together with its biotechnological application [59]. This heme-dependent property has now been found to be operating in many LAB, including several *Lactobacillus, Leuconostoc *and *Enterococcus *spp. [57,65,66]. Strictly speaking respiration is the coupling of a membrane potential to the reduction of oxygen and this only has been shown to operate in *Lactococcus lactis *subsp. *cremoris *MG1363 when grown on heme [67]. It is of interest to note that this respiration is so widely spread and appears to occur in food fermentations when there is a supply of heme-containing media. Remarkably, also the genome of *Oenococcus oeni *contains the genes for aerobic respiration but its functionality has not yet been tested [67].

By an elegant combination of genomics and expression studies, it has been shown that the *Lactococcus lactis *model strains IL1403 contains the genes for pili production that can be expressed and are involved in biofilm production [68]. Prior to this discovery such proteinaceous pili had only been described in the GI tract isolate *Lactobacillus rhamnosus *GG where they bind human mucus as well as have a set of other functions, *e.g*. immunogenicity [69,70]. The presence of these functional pili genes in strain IL1403 prompted comparative genomics studies that revealed their presence in various *Lactococcus lactis *strains, including the other model strain MG1363, the plant isolate KF147 (see above), and various other plant and human isolates [68]. The presence of pili production genes in dairy and plant strains suggests that this property is multifunctional and provides competitive advantage in various environments. Interestingly, by using a combination of proteomics and genomics, a functional pili cluster that enables mucus binding was also detected in another plant isolate, strain TIL448 [71]. Here, the genes for the pili production are located on a plasmid, suggesting horizontal gene transfer and proving a possible mechanism for the apparently wide spread of this novel function in dairy and plant lactococci.

## Functional genomics of LAB in human health

**Figure 3:**
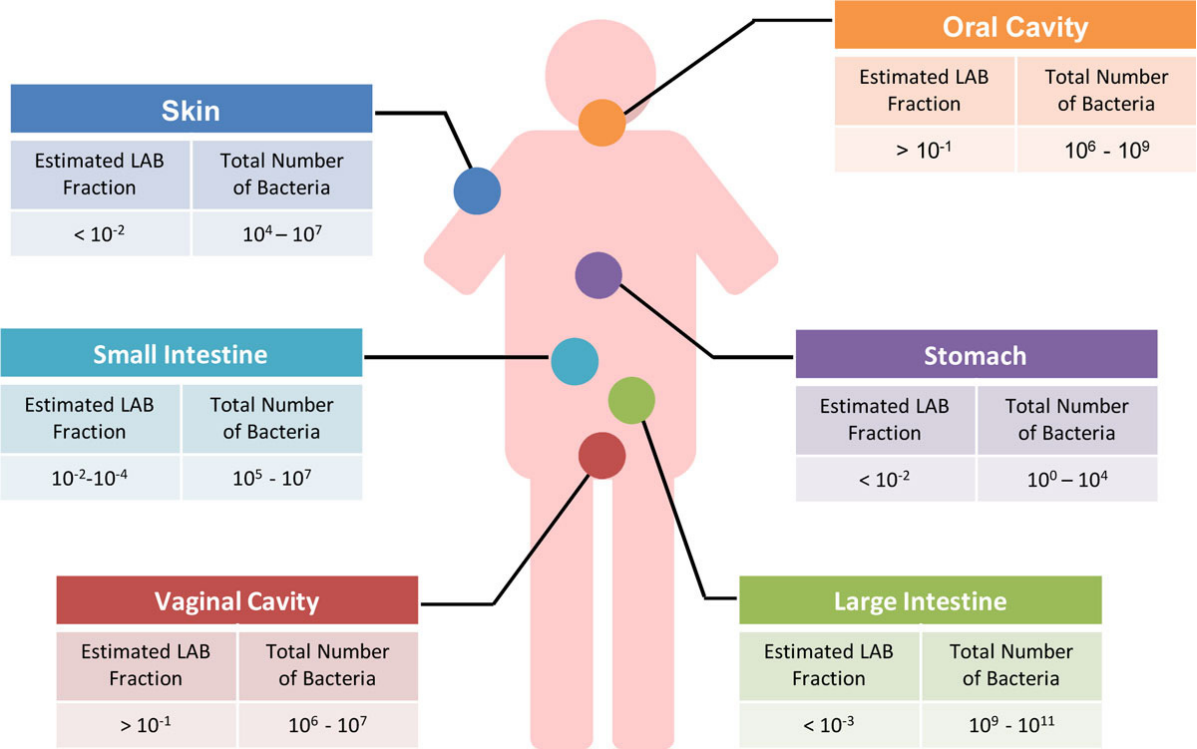
**Overview of the level of LAB in the different body sites**. The estimated LAB fraction is based on several complete and comprehensive phylogenetic and metagenomic datasets and the total number of bacteria per gram of homogenized tissue or fluid or square centimeter of skin [4,94,95,220,221].

The colonization of LAB in and on the human body has been well established and 16S rRNA-based phylogenetic studies have identified LAB at different body sites, such as the skin, oral cavity, GI tract, and vaginal cavity [72-77]. Further comprehensive phylogenetic and metagenomic characterizations of the human-associated microbiota using massive parallel sequencing, have extended this notion and identified the presence, level and genetic content of the various LAB in the microbial communities in the human body [4,5]. Based on these data it can be concluded that the number of total microbes varies considerably in the various body sites, as does the fraction of LAB (Figure 3).

The recent genome-based molecular inventories have shown that the fraction of LAB in the GI-tract is low and barely reaches over 1 % in only few persons (Figure 3). It is assumed that many of these LAB are passengers rather than endogenous inhabitants. Still, a detailed phenotypic and genomic characterization of strains from each LAB species is needed to clarify their role within the GI tract, since some LAB have a high intraspecies diversity and include both endogeneous and passenger strains. This has been confirmed in human feeding studies with marked *Lactococcus lactis*, showing unexpected survival of viable cells [78]. Moreover, a recent high fat feeding trial where fecal DNA was analysed using massive parallel sequencing, revealed the transit of *Lactococcus lactis, Streptococcus thermophilus *and *Pediococcus acidilacti*, which are components of dairy and meat starters [79]. However, based on genomic or sequence characteristics various LAB strains have found to be endogenous in human [56,73,75,80]. By far the highest fractions of LAB are found in the oral and vaginal cavities since the environment of these relatively open systems is more accessible than that of the human GI tract (Figure 3).

While our mouth as the port d'entrée of the GI tract is receiving a rather variable microbial load of mainly passengers, the vaginal cavity has a rather stable microbiota. This explains why the endogeneous vaginal LAB were found to be specifically associated with health [81]. This contrasts with the GI tract where most specific associations with health have been described for other members of the complex human-associated communities than LAB [82]. An exception is a recent metagenome study, where *Lactobacillus gasseri *was associated with the incidence of type 2 diabetes in a Swedish cohort [83]. However, this was not reproduced in another large type 2 diabetes cohort and the observed genes may have derived from passenger LAB [84].

As many of the genomes of human-derived LAB have been determined (Figure 2), we summarize the recent functional genomics studies of these strains below.

### The oral cavity

The mouth constitutes the first cavity from which food is introduced into the digestive tract. As an ecological habitat, it hosts hundreds of different bacterial species, including LAB, that are colonizing the teeth, the gum, the saliva and various locations on the tongue [4]. Teeth, as hard tissues, form an excellent surface for biofilm formation [85]. A dozen Lactobacilli are found to be the most prevalent LAB detected in the oral cavity (Figure 2) [86,87]. Metaproteomic analysis also confirmed the presence of Lactobacilli in the human saliva [88]. Some LAB have been used to restore healthy oral microbiota and the well-known probiotic *Lactobacillus rhamnosus *GG was shown to reduce the population of *Streptococcus mutans*, the common cause of caries [89]. Genomic and phenotypic characterization of oral isolates of *Lactobacillus rhamnosus *indicated that these were closely related to cheese isolates, suggesting that they may originate from food products [80]. However, genomic characterization of *Lactobacillus rhamnosus *strains isolated from dental pulp showed that these were unique and contained an additional set of approximately 250 unique genes [90]. These genes included those coding for the biosynthesis of exopolysaccharides that could be involved in biofilm formation, while others encoded transcriptional regulators and ferric iron ABC transporters. In the oral isolates of both studies, the *spa*CBA-*srt*C1 pilus gene cluster was lacking, suggesting that such trait is not essential for persisting in the oral cavity [80,90].

### The gastro-intestinal tract

Isolated or detected throughout the whole digestive tract, LAB only represent a minor proportion of gastro-intestinal microbial communities [73,91]. Typically, representatives of the *Lactobacillus/Enterococcus *group constitute 0.01-1.8% of the overall fecal microbiota, as shown by qPCR techniques [92]. Their abundance in the GI tract significantly ranges from less than 10^4 ^CFU/ml (small intestine) to 10^6 ^CFU/g (faeces) (Figure 3) [73,74,93-95]. The human small intestine was shown to harbour a diverse population of Streptococci [96]. However, sequence analysis of the rRNA gene does not allow determining whether these detected LAB strains are endogenous or transient. Up to date, more than 20 LAB species have been detected in the digestive tract (Figure 2). Some of these are consumed as probiotics, such as *Lactobacillus plantarum, Lactobacillus casei *or *Lactobacillus rhamnosus *[8,10,97]. Others are present in the mouth where they may be derived from food or be endogenous (see above). This suggests that some of the LAB isolated from the GI tract may in fact originate from food or the oral cavity [96,98,99].

Detailed comparative and functional genomic characterization of human LAB isolates may provide answers whether they are endogenous or transient, as well as generate a better understanding of their ecological fitness, their adaptation, and their role in their dedicated niche. The first of these studies related to *Lactobacillus johnsonii *and *Lactobacillus gasseri*, which were genomically characterized ten years ago (Table 1). Genomic data complemented with experimental work provide evidence for the ecological adaptation and fitness of *Lactobacillus gasseri *to the GI tract, as recently reviewed [100]. Transcriptomic analysis of *Lactobacillus johnsonii *NCC533 identified a number of genes that could relate to its persistence within the intestinal tract [101]. The isolation and sequencing of intestinal LAB along with LAB from other sources has allowed us to compare strains and to determine the diversity of each species from an ecological but also evolutionary perspective. In a recent comparative genomic study, the examination of 100 *Lactobacillus rhamnosus *isolates showed possible correlations between ecological fitness, phenotypic traits and genomic modifications [80]. The intraspecies diversity in *Lactobacillus rhamnosus *was mostly concentrated in 17 lifestyle islands. Compared to *Lactobacillus rhamnosus *food isolates, a subset of GI tract isolates harbored more prevalently genes associated with specific carbohydrate pathways (fucose metabolic genes), host adhesion (mucus-binding SpaCBA pilus gene cluster), defence and immunity system (CRISPR system) and biofilm formation (exopolysaccharide cluster). These are likely to provide an improved capacity to colonize and persist in the GI tract [80]. Intestinal *Lactobacillus rhamnosus *isolates were shown to be resistant to bile, whereas isolates from dairy niches for example were generally less bile-resistant [80]. Two other closely related species *Lactobacillus casei *and *Lactobacillus paracasei *shared some lifestyle islands with *Lactobacillus rhamnosus *that were syntenous [102,103]. Using hybridization arrays and multilocus sequence typing, the genomic diversity of *Lactobacillus salivarius *was studied [104]. In line with findings in other LAB, the intraspecies diversity was found to be concentrated on 18 chromosomal regions that included gene clusters encoding for the production of exopolysaccharides [104]. An important fitness factor with applied potential is the capacity to produce a broad host-range bacteriocin that allowed *Lactobacillus salivarius *to outcompete *Listeria monocytogenes *[105]. In addition to chromosomal variations, the presence of plasmids and other mobile elements are playing an important role. One remarkable example contributing to intraspecies diversity is the presence of megaplasmids in some *Lactobacillus salivarius *strains. *Lactobacillus salivarius *subsp. *salivarius *UCC118 harbors the megaplasmid pMP118 (242 kb in size) [106]. Further analysis of two other subspecies identified other megaplasmids with a different size, suggesting a possible role in ecological adaptation [106].

Some species such as *Lactobacillus reuteri *are specialized to one particular host. *Lactobacillus reuteri *is also commonly in different human body sites, *i.e*. breast milk, GI tract, vagina but it is also found in other vertebrates [107,108]. Work on the *Lactobacillus reuteri *species revealed that strains have distinctly evolved between different hosts. Gut isolates from different mammals, *i.e*. rodents and humans have distinct genetic signatures. This may be explained by the fact that the anatomical differences between human and rodent gut resulted in different colonization strategies [109]. The host specialization observed among *Lactobacillus reuteri *strains results from similar genetic mechanisms as in other symbiotic bacteria [109]. The role played by transposases in the genome dynamics between rodent and human isolates differs. The genomes of *Lactobacillus reuteri *human gut isolates tends to be smaller with higher number of pseudogenes [109], as previously reported in other host-dependent bacteria [110]. In contrast with the *Lactobacillus reuteri *strains, where it was shown that strain differ according to the host, comparative genomic analysis showed that the human gut strain *Lactobacillus ruminis *ATCC 25644 is highly similar to the bovine isolate *Lactobacillus ruminis *ATCC 27782 [111]. They, however, significantly differ from the closely related *Lactobacillus salivarius *(Figure 3). *Lactobacillus acidophilus *and *Lactobacillus helveticus *are closely related (Figure 2). However, *Lactobacillus helveticus *is typically more specialized to the dairy environment compared to the gut-adapted *Lactobacillus acidophilus*, which has conserved more biological functions. In the *Lactobacillus helveticus *genome, adhesion factors, such as mucus-binding proteins, are absent along with a narrower gene repertoire encoding for PTS transporters [112,113].

Genome sequences of LAB provided a basis to identify the secretome and interactome of LAB found in the human GI tract. Within the *Lactobacillus casei *group, the respective LPXTG protein-encoding gene repertoires of *Lactobacillus rhamnosus, Lactobacillus casei *and *Lactobacillus paracasei *shared several similarities [102]. Among others, pilus gene clusters were identified. However, only in *Lactobacillus rhamnosus*, the functionality and expression of one of the gene cluster encoding mucus-binding pili (*spa*CBA-*srt*C1) has been so far demonstrated [69,114]. This single and outstanding trait contributes to the highly efficient adhesion of *Lactobacillus rhamnosus *GG to the intestinal mucosa [69]. Within the *Lactobacillus rhamnosus *species, pilus-associated genes were significantly more present in intestinal isolates (56 %) compared to dairy isolates (13 %) [80]. Genome-wide analysis of *Lactobacillus salivarius *UCC118 identified 108 predicted secreted proteins, including 10 sortase-anchored proteins. Gene deletion of sortase and one sortase-anchored protein significantly reduced the epithelium-binding ability of the strain UCC118 [115]. A recent review discussed the central role of sortases and LPXTG proteins for LAB, especially for the ones found in the GI tract [116]. Interestingly, some *Lactobacillus ruminis *strains, *i.e*. ATCC 27782, also possess a set of genes encoding for a complete and functional flagellar apparatus, *i.e*. 45 flagellar genes, providing motility [117]. The discovery of motile commensal LAB suggests unique and uncovered impact on the gut ecology in terms of host signaling and colonization. In the intestinal *Lactobacillus gasseri *ATCC 33323, among the 271 predicted cell surface proteins, at least 14 mucus-binding proteins were identified [118], suggesting a potential role in adherence with the intestinal mucosa. In *Lactobacillus acidophilus *L-92, the attachment to epithelial cell lines altered the expression of 78 genes, *i.e*. membrane proteins, transporters and regulators [119]., Comparative proteomic analysis led to the identification of 18 proteins with potential adhesive properties, including surface-layer protein A. Further work showed that the latter protein has a central role in the adherence of *Lactobacillus acidophilus *L-92 to epithelium [120]. Moreover, one of the well-characterized surface-layer proteins, SlpA of *Lactobacillus acidophilus *NCFM, was found to bind to the DC-SIGN receptor of dendritic cells, indicative of a role in intestinal signaling [121,122].

A number of similarities in terms of response to the GI environment have been observed among gut-isolated LAB species and relate among others to metabolic re-routing, cell wall modifications or activation of resistance/stress mechanisms. The mechanisms by which these genes are induced when LAB are in the human gut are not fully comprehended. Specific attention has been given to the exposure to bile salts and acids as during the transit (and eventual colonization) in the GI tract, LAB are exposed to these environmental stimuli. Recent proteomic and transcriptomic analysis of the intestinal *Lactobacillus rhamnosus *strain GG under bile stress revealed the activation of numerous genes related to cell wall functions and possibly operate as a stimulus for adherence in the intestinal tract [123]. *Lactobacillus rhamnosus *strain GG also generated a specific response towards acid environments, as examined by proteomic analysis [124]. Similarly, in *Lactobacillus casei *BL23, 52 proteins showed an altered expression under bile stress, and these were predicted to be involved in general stress response, cell wall functions and also carbohydrate metabolism [125]. Remarkably, in *Lactobacillus acidophilus*, glycogen metabolism was found to be associated with bile resistance [126]. Apart from these laboratory studies also a series of model animal and human studies have been reported. An *in vivo *expression technology (IVET) study in *Lactobacillus plantarum *WCFS1 identified a set of 72 genes that were induced when transiting the GI tract of mice [127]. These mainly include genes associated with carbohydrate metabolism, biosynthetic pathways and transport and also four genes potentially relating to host interactions, *i.e*. cell wall anchor proteins [127]. Reciprocally, *Lactobacillus plantarum *WCFS1 cells triggered the expression of over 400 genes in the mucosa of the human small intestine [128,129]. A mouse study further addressed the transcriptional responses of *Lactobacillus plantarum *to different dietary regimes [130]. Finally, the transcriptional responses to *Lactobacillus plantarum *WCFS1 in mice and human were described in a detailed comparative study that revealed high level similarities between those systems [131]. The transcriptomic profile of *Lactobacillus plantarum *WCFS1 was also found to be modified upon exposure to p-coumaric acid, a component present in vegetables or fruits, possibly signaling *Lactobacillus plantarum *to its entry to the digestive tract [132]. Similarly, the transcriptional response of *Lactobacillus plantarum *to bile was also investigated, revealing a set of genes whose expression is bile-inducible [133]. Within the *Lactobacillus plantarum *species, strains have different bile sensitivity, *i.e*. showing either resistance (strain 299V) or sensitivity (strain LC56) [134]. Comparative proteomic analysis of three different strains led to the identification of 13 proteins related to bile resistance mechanisms [134]. In addition, alteration of genes associated with cell surface proteins and metabolism suggests that *Lactobacillus plantarum *underwent adaptation when exposed to the murine tract [135]. In intestinal isolates of *Lactobacillus reuteri*, a total of 28 genes were shown to be induced under bile salt exposure and proteomic analysis indicated that the encoded proteins were associated with metabolic pathways, stress-induced response and also pH homeostasis, which possibly relate to resistance mechanisms of *Lactobacillus reuteri *to bile salt stress [136]. A similar mechanistic response was observed when exposed to acids [137]. Mice studies showed that the transcriptome of *Lactobacillus johnsonii *NCC533 is changing throughout the GI tract, suggesting specific responses to each of the GI sites [138]. Using a mouse model, it was found that 174 *Lactobacillus johnsonii *NCC533 genes were expressed *in vivo*, including EPS-associated glycosyltransferase genes and PTS transporters [101].

In conclusion, LAB when present in the GI-tract express a number of common characteristics that relates their adaptation. These could be summarized as follows: *i*. a large repertoire of genes encoding transporters (ABC, PTS or permeases) to optimally utilize nutrients available in the gut niche, *ii*. the presence of genes associated with acid and bile resistance, *iii*. a wide range of genes promoting interactions and signaling with the host, such as pili that contain mucus-binding proteins.

### The vaginal cavity

LAB members constitute a dominant proportion (~80%) of bacteria inhabiting the vaginal cavity of healthy women [139] and are consistently detected in healthy vaginal microbiota from patients of different ethnic groups and/or living in different geographical locations [139-143]. Four main bacterial species were typically identified: *Lactobacillus crispatus, Lactobacillus iners, Lactobacillus jensenii *and *Lactobacillus gasseri *along with, at lesser extent, some other lactobacilli, such as *Lactobacillus acidophilus, Lactobacillus ruminis, Lactobacillus rhamnosus *or *Lactobacillus vaginalis *[139,144-146]. The high abundance of LAB is strongly associated with healthy vagina, whereas a low abundance of LAB, *i.e*. alteration of the vaginal microbiota, was more prevalent in women with a medical condition, *i.e*. bacterial vaginosis (BV) [140,145,147]. The beneficial roles of LAB in preserving a healthy vagina include the maintenance of acidic vaginal pH [148], the prevention of infections by producing bacteriocins, hydrogen peroxide and acids, but also by signaling to the host [148-150]. The understanding of the vaginal microbiota composition not only contributes to the comprehension of the ecology of this habitat in health and disease but also offers avenues towards the development of better diagnostic and therapeutic solutions [147,151,152].

Four LAB species are predominantly detected in human vagina (*Lactobacillus crispatus, Lactobacillus gasseri, Lactobacillus iners *and *Lactobacillus jensenii*) but co-dominance between LAB species is seldom [142]. This indicates that each vaginal species may harbor genes that relate to (unique) adaptation signatures and allow the non-symbiontic persistence and colonization regardless of the presence of other LAB members [153]. Interestingly, these LAB genomes also showed to be significantly smaller and contained a lower GC content than other LAB genomes, suggesting a loss of non-essential genes towards a vaginal adaptation [153].

One of the most studied vaginal LAB is the *Lactobacillus iners*. Remarkably, strains from the *Lactobacillus iners *species have a relatively small genome compared to the LAB, *i.e*. ~1.3 Mb for *Lactobacillus iners *AB-1 genome [154] and its intraspecies diversity is peculiarly low [143]. In line with its genome size, *Lactobacillus iners *is not able to biosynthesize many vitamins, cofactors and amino acids, while compensating these metabolic limitations by the presence of numerous genes encoding transporters [154]. When compared to *Lactobacillus crispatus, Lactobacillus gasseri *and *Lactobacillus jensenii, Lactobacillus iners *carries a variety of unique genes encoding ABC transporters [153]. The poor metabolic and biosynthetic capabilities illustrate its strong dependency to the host niche, from where *Lactobacillus iners *acquires most of its nutrients. This may also explain why this species is rarely detected in other ecological niches that are more demanding in terms of metabolic capabilities [155,156]. *Lactobacillus iners *is lacking numerous transcriptional regulators or integral membrane proteins [153]. The detailed mechanisms involved the persistence of *Lactobacillus iners *in the vagina remain unclear. However, a number of genes encoding potential adhesins (a total of 11 LPXTG proteins) were identified in *Lactobacillus iners *AB-1 [154], along with genes encoding fibronectin-binding type adhesins [157], indicating that interactions occur between the bacterial cells and the vaginal tissues. Such association (lactobacilli-epithelium) promotes exclusion of pathogens [158], as shown with the displacement of biofilms formed by *Gardnerella vaginalis *[159]. In addition, *Lactobacillus iners *AB-1 is able to use mucin as a carbon source, which is clearly beneficial for persisting in a mucosal niche (vagina) [154]. Interestingly, the genome of *Lactobacillus iners *AB-1 contains a gene (LINAB_0216) that encodes a cytolysin [154]. This gene is also found in other *Lactobacillus iners *isolates and its product is similar to cholesterol-dependent cytolysins produced in species such as *Streptococcus *or *Gardnerella*, [160]. However, its function in *L. iners *is unclear, *i.e*. attachment to host tissues, antimicrobial activity or pathogenesis [143,160]. A recent meta-RNA-seq based study showed that during a BV episode *Lactobacillus iners *AB-1 modified the expression of genes encoding the CRISPR-*cas *system, the cholesterol-dependent cytolysin and the mucin and glycerol transporters [81]. This underlines adaptive mechanisms towards the persistence of *Lactobacillus iners *in changing vaginal microbiota, *i.e*. change of nutrient use (mucin and glycogen) and protection against bacteriophages [81]. The overexpression of the cholesterol-dependent cytolysin by *Lactobacillus iners *during BV appeared to have a detrimental role towards the host [152]. Based on genomic and transcriptomic data, *Lactobacillus iners *was found to be specifically adapted the vaginal niche under different conditions, *i.e*. healthy or non-healthy vaginal microbiota. This remarkable adaptation suggests a strong association of *Lactobacillus iners *with the host, possibly contributing to maintaining a healthy microbiota, though its role in BV needs to be further examined.

In contrast with the *Lactobacillus iners *species, strains of all three other vaginal LAB, *Lactobacillus crispatus, Lactobacillus gasseri and Lactobacillus jensenii *are also found in other ecological niches than the vagina (Figure 2). Intestinal *Lactobacillus gasseri *isolates have genotypic traits beneficial for persistence and colonization in the gut (see above) [118]. Comparative genomic analysis identified a series of species- and/or niche-specific gene sets mostly consisting of different ABC transporters and regulators and in some cases toxin-antitoxin systems or cell envelope proteins [153]. However, no clear vaginal gene sets were defined in *Lactobacillus crispatus, Lactobacillus gasseri and Lactobacillus jensenii*. Vaginal strains of *Lactobacillus crispatus *have a larger genome than other strains of this species, possibly resulting from an abundance of IS-encoded transposases [153].

Apart from the four dominant LAB species that are recurrently detected in healthy vaginal microbiota, also other *Lactobacillus *spp., can be found and show, in some cases, unique patterns in both phenotypes and genomes (Figure 2). In a recent study, vaginal *Lactobacillus rhamnosus *isolates were compared with the *Lactobacillus rhamnosus *strain GG at both genomic and phenotypic level [80]. Four main genotypic/phenotypic traits were highlighted: the lack of mucus-binding pili, their bile resistance (100% of all isolates), an altered or deficient CRISPR-*cas *system compared to strain GG and some metabolic capabilities similar to food isolates. It was hypothesized that vaginal LAB may have originated from food environments or the oral cavity and survived through the gastro-intestinal tract (bile resistant, antimicrobial activity), before colonizing the vaginal cavity [80]. The loss of the pilus gene cluster indicates that it is not beneficial for *Lactobacillus rhamnosus *in the vaginal cavity. This is consistent with genomic data on other vaginal LAB, such as *Lactobacillus iners, Lactobacillus gasseri *or *Lactobacillus crispatus*, with genomes that does not contain such cluster. Recent work on other LAB, *i.e. Lactobacillus plantarum*, showed that the vaginal adhesion of the bacterial cells is sortase-dependent and therefore relies on LPXTG anchor proteins that likely do not form pili [161]. Similar mechanisms may occur as well in other LAB, such as *Lactobacillus rhamnosus*. No other studies on vaginal *Lactobacillus rhamnosus *genomics have been reported but it seems that only a subset of the *Lactobacillus rhamnosus *species may be able to colonize the vaginal cavity. Most clinical trials using *Lactobacillus rhamnosus *strains showed promising results [162,163]. However, each strain within the species appear to have a distinct ecological fitness and intestinal *Lactobacillus rhamnosus *strain GG with a pheno-genotype different from vaginal isolates, was poorly colonizing the vagina cavity, indicating that it lacks a number of genes promoting its ecological fitness to the vaginal cavity [164].

### Other body sites and clinical cases

In general, LAB are considered to be safe and many species are on the list of Qualified Presumed Safety (QPS) of the European Food Safety Authority [165]. This does not apply to *Enterococcus faecalis *and *Enterococcus faecium*, two species of enterococci that have been and are used as starters in various food fermentations as well as marketed as probiotics (Figure 2) [166]. These enterococci emerged as the leading causes of antibiotic-resistant infection of bloodstream, urinary tract and surgical wounds [167]. However, most if not all human are carrying these *Enterococcus *spp. in their GI tract and it has been suggested that enterococci may have been ubiquitous colonizers of the gut since the early Devonian period, *i.e*. 400 million years ago [168]. Comparative genomic studies have now shed light on how such normal colonizing species may have developed into a major group of pathogens. It appeared that the genomes of hospital adapted enterococcal strains consist of over 25 % of mobile elements, have lost CRISPR-*cas *systems that limit horizontal gene transfer, and have accumulated multiple antibiotic resistance and virulence traits [168]. It has been proposed that the introduction of antibiotics approximately 75 years ago and their widespread use in both human and veterinary medicine promoted the rapid evolution of the present epidemic hospital-adapted lineage not from human commensals but from a population that included animal strains [168]. There is some apparent disagreement about the moment of divergence between the commensal and hospital lineages of enterococci (300,000 versus 3000 years ago) [168,169]. However, it is tempting to assume that this occurred after the transition of the hunter-gatherer, possibly at a time of increasing urbanization of humans, development of hygienic practices, and domestication of animals as has proposed to contribute to the ecological separation of these lineages [168] (Figure 1). Interestingly, a comparative genomic study indicated that *Enterococcus *spp. and pathogenic Streptococci shared more gene families than did the genomes from non-pathogens, such as other LAB [170].

Inspection of the present QPS listing reveals that some LAB have incidental cases where they are implicated in non-nosocomial and other clinical infections. This has been described previously for *Lactobacillus rhamnosus *and has been recently reviewed [171]. However, the increased intake of *Lactobacillus rhamnosus *GG did not lead to an increase in bacteremia cases [172]. Hence, EFSA concluded that clinical infections especially of *Lactobacillus rhamnosus*, should be closely monitored [165]. This also relates to an increasing number of reports that imply LAB in other body sites than the canonical caveats (Figure 3). These include strains of *Lactococcus lactis, Leuconostoc lactis, Lactobacillus casei, Lactobacillus paracasei *and *Pediococcus *sp. [165]. The number of reports linking *Lactococcus lactis*, often the subsp. *cremoris*, to clinical cases is increasing. Recent studies include the isolation of *Lactococcus lactis *from human brain or neck abcesses or bovine mastitis [173-175]. It should be remembered that *Lactococcus lactis *(then appropriately termed *Bacterium lactis) *was the first bacterium grown as a pure culture by Joseph Lister in 1878. Ironically, Lister compared the fermentation process with an infection process in his attempts to illustrate the cause of infectious disease in humans [176]. It can be expected that further comparative and functional genomic studies of clinical, food and other LAB isolates will be instrumental in understanding the adaptations to the human body as well as assessing the safety of LAB used in the food or pharmacy industry.

## Evolutionary LAB genomics

### Adaptation and horizontal gene transfer

It is generally believed that plant material is the archetype source of the dairy LAB, though some inoculation from the dairy cow and its milk is also possible (Figure 1). Recent culture-independent analysis of the foliar microbiome, which is rapidly developing and the dairy cow's teat showed LAB to be present in both environments [177,178]. Hence, detailed genomic analysis is needed to distinguish between the sources of the dairy LAB. Comparing the genome of the plant isolate *Lactococcus lactis *subsp *cremoris *KW2 with the dairy strains showed remarkable similarities apart from the large 21-gene cluster coding for the biosynthesis of wall techoic acids that is partially absent or truncated in the model strain MG1363 or the dairy starters SK11, UC509.9 or A76. In contrast to the dairy starters, the plant strain KW2 does not contain any plasmids or IS sequences. This substantiates the earlier suggestions that these mobile elements are recent acquisitions by horizontal gene transfer. Moreover, the presence of the gene cluster for the wall techoic acid production seems to be a plant adaptation as it is also found in *Lactococcus lactis *subsp. *lactis *KF147 isolated from mung bean sprouts that has been studied extensively as a non-dairy model for lactococci [23]. This strain KF147 has one of the largest genomes, shows high identity and synteny to the genome of *Lactococcus lactis *subsp. *lactis *IL1403 but contains a variety of plant adaptations that have been lost in the dairy starter of this taxon [23,179]. Hence, for Lactococci there is ample evidence that plants are the sources of the dairy strains (Figure 2).

The genome *Lactobacillus iners *AB-1 is the smallest among the LAB (Table 1) suggesting that important gene loss occurred in that species towards the specialization to one unique ecological habitat, *i.e*. vaginal cavity. The genome size reduction possibly reflects the dependency of vaginal LAB to their host, as previously reported in other symbiotic bacteria, such as *Candidatus Tremblaya *princeps (genome size of 139 kb) [180]. The limited coding capacities of *Lactobacillus iners *do not only reflect a remarkable ecological-driven specialization to the vaginal host but also a strong dependency to this habitat. The high number of genes associated with DNA repair, RNA modification and the alteration of a number of metabolic pathways clearly underline how most of these vaginal lactobacilli rely on the host for surviving and persisting. There is a potential mutualistic relationship between the host and the vaginal LAB. The host provides a stable environment, from where vaginal LAB can utilize nutrients (mucin, glycogen) or by-products from other inhabitants. In return, vaginal lactobacilli are warrant of the maintenance of a healthy vaginal microbiota. Although *Lactobacillus iners *has been reported in rare clinical cases [155], these may constitute evolutionary dead-ends that are usually not associated with any adaptation traits.

As detailed in the first large scale comparative genomic study, most LAB are phylogenetically closely related (Figure 2) but mainly differ by the gain of novel genes or the loss/decay of ancestral genes [19]. In addition, the number of pseudogenes is highly variable among LAB, *i.e. S. thermophilus *CNRZ1066 (182 pseudogenes) [181] or *Pediococcus pentosaceus *ATCC 25745 (19 pseudogenes) [19]. The presence of plasmids or megaplasmids in some strains are also of interest, since they may carry additional genes involved in metabolic pathways, production of bacteriocins and bile salt hydrolase. Two striking examples are: the co-existence of 8 plasmids in *Pediococcus claussenii *ATCC BAA-344 [182] and the presence of a 242-kb megaplasmid pMP118 in *Lactobacillus salivarius *UCC118 [106]. In addition, horizontal gene transfer further contribute to genus and species diversification, as previously reported in *Lactobacillus acidophilus, Lactobacillus casei, Lactobacillus delbrueckii *subsp. *bulgaricus *and *Lactobacillus johnsonii *[103,183-185]. Significant differences observed in LAB genomic features give primary evidence for possible ecological adaptation and specialization: genome size (coding capacities), pseudogenes or plasmids (Table 1). Only a further detailed examination of these genomes may highlight gained, duplicated, decayed or lost gene sets that are encoding biological functions relating to one particular ecological context. The role played by transposases in the genome dynamics between rodent and human isolates differs. The genomes of *Lactobacillus reuteri *human gut isolates tends to be smaller with higher number of pseudogenes [109], as previously reported in other host-dependent bacteria [110].

### Applied LAB genomics

The use of functional and comparative genomics has greatly enhanced a variety of applications. First, there is the issue of strain identity and protection. Many manufacturers of LAB starters or producers that market LAB as probiotics, have started to characterize their strains by complete genomic analysis. While supporting rapid strain characterization, this is also instrumental in strain mining and speedily selecting specific properties. Moreover, safety, administrative and legal processes can be supported by genome sequences and LAB strains of competitors can be benchmarked. With respect to safety, one should realize that knowledge of a genome sequence does not make a strain safe or not. However, lessons learned from the adaptation of notably *Enterococcus *strains discussed above could be helpful in further predicting safety of LAB.

The rapid implementation of next generation sequencing technologies for comparative genome analysis has allowed for several well-known commercial strains to be made public. It was recently shown that *Lactobacillus casei *strains marketed in Yakult and Actimel products were found to contain only a few dozen single nucleotide polymorphisms (SNPs) and a prophage [186]. This approach also showed that *Lactobacillus rhamnosus *GG isolated from several products was highly stable [114]. A new genomics approach that is only possible by the rapid advances in sequencing technology is capitalizing on genomic resequencing approaches. In a first published example *Lactococcus lactis *NZ9000, containing the *nisRK *two-component system genes that are used in conjunction with the nisin-controlled expression system, was mutated to increase expression of a variety of membrane proteins [17,187]. The genomes of the resulting 3 strains were compared and found to carry notably SNPs in the sensor NisK gene [17]. This coupling of adaptive evolution and high throughput sequencing has been used in many other studies with LAB, *e.g*. experimental evolution of *Lactobacillus plantarum *when exposed to the murine digestive tract [135]. A recent report describes an elegant study with the plant isolate *Lactococcus lactis *KF147 (see above) that propagated for 1000 generations in milk resulting in faster growth and biomass yields [188]. Three of the resulting strains were resequenced and found in two of the cases to have lost the conjugative transposon needed for growth in plants (see above). In the rest of the genome only few (6-28) mutations were detected in various genes, including those involved in amino acid production and transport. Remarkably, the strain with most mutations also contained a mutated *mutL *gene involved in mismatch repair and believed to increase the mutation frequency [188]. This example illustrates not only the power of experimental evolution and the used sequencing technology but also highlights the domestication process of a plant strain to the dairy environment.

A final but appealing approach where applied genomics has been used is the in the selection for *Lactococcus lactis *strains [189]. Cells of the strain MG1363 were mutagenized and serially propagated in water-in-oil emulsions to allow for selection of strains with increased biomass yield. One of the resulting strains coupled an increased biomass to slightly different growth kinetics and the conversion from homolactic into a mixed acid fermentation. Genomic resquencing revealed a SNP mutation in the *ptnC *gene, encoding a component of the glucose PTS transport system. The phenotype of this mutant is explained by decreased glucose uptake rates, resulting in less acidification and higher yields without pH control. A series of revertants were also isolated that upon genomic resequencing were found to contain an IS905 copy inserted in front of the *ptnABCD *operon, resulting in upregulation of the glucose PTS transport [189]. While these experiments generated further insight in fundamental aspects of the adaptation processes they also represent the proof of concept on how to use high throughput screening and sequencing allowing rapid analysis of the results. The examples of applied genomics described here are only a few of the possibilities that can be envisaged. Notably, strain optimization in combination with genomic re-sequencing will be a highly useful tool for improving starter strains or LAB marketed as probiotics. As natural or induced mutations do not lead to genetically modified organisms, the generated and improved strains can be used immediately for food or pharmaceutical applications.

## Concluding remarks

**Figure 4:**
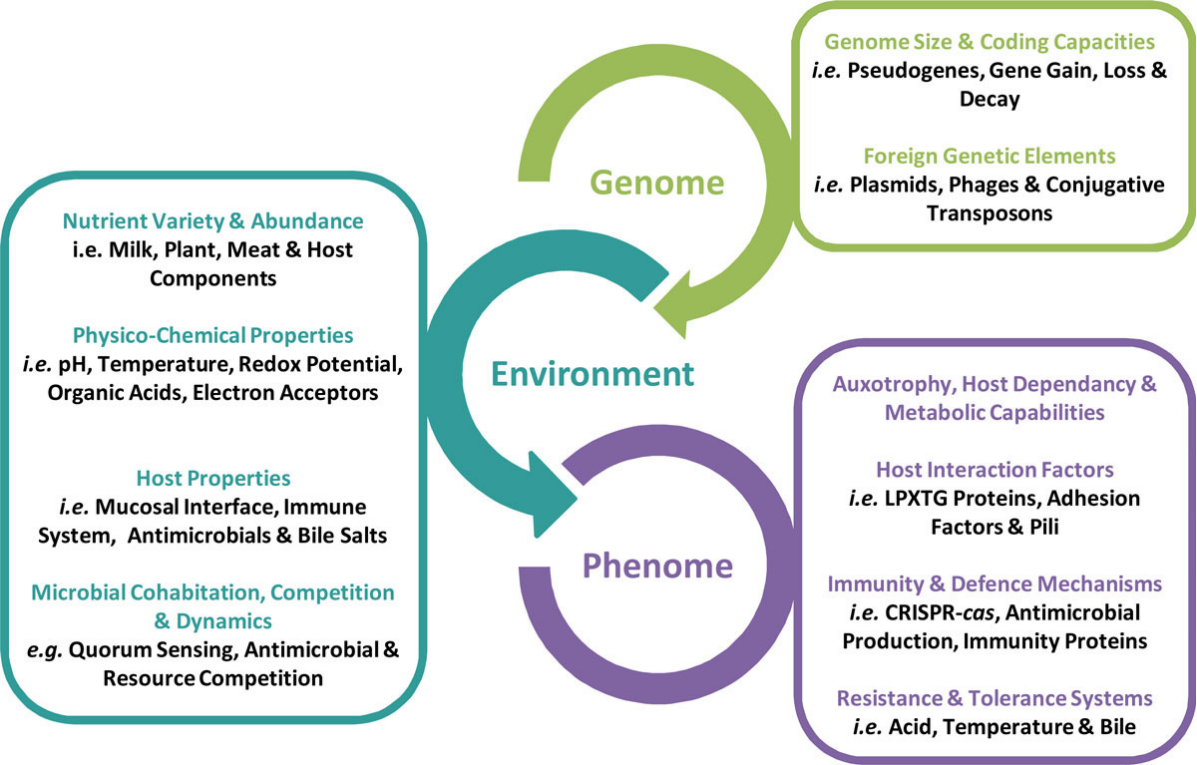
**Genome, habitat and phenome - a summary overview**.

Benefiting from the rapid development of next generation sequencing techniques, multiple genome sequencing projects on LAB were initiated since the beginning of the millennium. The data available up to now provide a comprehensive view on the complexity of the heterogeneous LAB group (Figure 2). Detailed comparative analysis of these genomic data emphasized the remarkable diversity within the LAB group at numerous taxonomic levels, *i.e*. order, family, group, genus and even species. This diversity results from the interactions between genome and environment as is schematically depicted (Figure 4). The abundance and variety of nutrients available in a habitat has a direct impact of the catabolic and biosynthetic properties of LAB. In many LAB species, the loss of metabolic genes is compensated by genome enrichment in genes encoding for transporters (ABC or PTS systems), allowing LAB to use nutrients and by-products from their niche. This specialization is evident from genome size reduction, presence of pseudogenes, and genome decay. Still, other LAB species or strains maintain a broad ecological flexibility, which may cause a high resilience to drastic environmental changes.

Because LAB are heterotrophs they have developed intimate interactions with plants and, most likely later, with animals and humans (Figure 1). Host-associated LAB contain a large and diverse repertoire of interaction proteins to adhere and signal to the host. It is tempting to speculate that the GI tract, as the site where plants enter the animal body, has played an important role in this evolutionary process. LAB adapted to the food environment may not require interaction with any host and therefore would generally possess a distinct repertoire of cell surface proteins. Thus, alternative surface proteins may be involved in the interactions between LAB and food constituents as compared to the interplay with the host mucosa [190]. Horizontal gene transfer appears a major driver of the genomic diversity and plasticity, affecting genome size and the acquisition of new genes. Plasmids of different sizes (up to mega-plasmids) and conjugative transposons have found to be involved in gene gain and loss.

Surviving in a niche also means to compete with other microbes and to defend against other inhabitants, including bacteriophages. The controlled production of organic acids and antimicrobials is a highly effective strategy in this microbiological warfare. Moreover, LAB harbor CRISPR-*cas *systems to protect from bacteriophages and other foreign DNA. It seems that the loss of these defense systems may promote the promiscuous transfer of various traits, including antibiotic resistance or virulence factors. Finally, tolerance and resistance systems to endure physico-chemical properties, such as temperature, acid, salt or bile salts, are essential for LAB living in foods, the GI tract or other harsh environments.

The area of host-microbe, microbe-microbe and microbe-molecule interaction is a highly relevant and timely theme, notably in view of the rapidly expanding interest in the human GI tract [191]. It may be expected that the insight worked out for LAB may serve as model for other microbes. Moreover, as many LAB have immediate application potential, these systems also may result in improved or novel strains or processes, as seen for the discovery of peptide-based quorum sensing in *Lactococcus lactis *[192]. Some of the models with impact at various levels include the CRISPR-*cas *system discovered in *Streptococcus thermophilus *[193], the communication of *Lactobacillus plantarum *with the human host [129], the production of host-interacting pili in *Lactobacillus rhamnosus *[69], the evolution of metabolic strategies in *Lactococcus lactis *[189] or the finding of a novel metal-depending lactate racemase in *Lactobacillus plantarum *that is widely distributed [194]. The discovery of these models has relied for a large part on functional genomics, stressing the importance of this approach in LAB. This provides a promising outlook for the future where soon all LAB species will be characterized at the genomic level, many strains will have been re-sequenced, and functional and applied genomics are implemented in academic and industrial environments, resulting in the further advancement of science and improvement of the quality of life.

## List of abbreviations used

BV, bacterial vaginosis; DS, direct submission; GI, gastro-intestinal; GMO, genetically-modified organisms; LAB, lactic acid bacteria; NSLAB, Non-Starter LAB.

## Competing interests

The authors declare that they have no competing interests.

## References

[B1] Tannock GW Normal microflora: an introduction to microbes inhabiting the human body 1995 Springer

[B2] Vaughan EE Heilig HGHJ Ben-Amor K de Vos WM Diversity, vitality and activities of intestinal lactic acid bacteria and bifidobacteria assessed by molecular approaches FEMS Microbiol Rev 2005 29 477 490 10.1016/j.fmrre.2005.04.009 16125009

[B3] Zoetendal EG Rajilić-Stojanović M de Vos WM High-throughput diversity and functionality analysis of the gastrointestinal tract microbiota Gut 2008 57 1605 1615 10.1136/gut.2007.133603 18941009

[B4] Human Microbiome Project Consortium Structure, function and diversity of the healthy human microbiome Nature 2012 486 207 214 10.1038/nature11234 22699609 PMC3564958

[B5] Qin J Li R Raes J Arumugam M Burgdorf KS Manichanh C Nielsen T Pons N Levenez F Yamada T A human gut microbial gene catalogue established by metagenomic sequencing Nature 2010 464 59 65 10.1038/nature08821 20203603 PMC3779803

[B6] Sekelja M Rud I Knutsen SH Denstadli V Westereng B Naes T Rudi K Abrupt temporal fluctuations in the chicken fecal microbiota are explained by its gastrointestinal origin Appl Environ Microbiol 2012 78 2941 2948 10.1128/AEM.05391-11 22307311 PMC3318845

[B7] Frese SA MacKenzie DA Peterson DA Schmaltz R Fangman T Zhou Y Zhang C Benson AK Cody LA Mulholland F Molecular characterization of host-specific biofilm formation in a vertebrate gut symbiont PLoS Genet 2013 9 e1004057 10.1371/journal.pgen.1004057 24385934 PMC3873254

[B8] Saxelin M Tynkkynen S Mattila-Sandholm T de Vos WM Probiotic and other functional microbes: from markets to mechanisms Curr Opin Biotechnol 2005 16 204 211 10.1016/j.copbio.2005.02.003 15831388

[B9] Petschow B Dore J Hibberd P Dinan T Reid G Blaser M Cani PD Degnan FH Foster J Gibson G Probiotics, prebiotics, and the host microbiome: the science of translation Ann N Y Acad Sci 2013 1306 1 17 10.1111/nyas.12303 24266656 PMC4013291

[B10] Tannock GW A special fondness for lactobacilli Appl Environ Microbiol 2004 70 3189 3194 10.1128/AEM.70.6.3189-3194.2004 15184111 PMC427720

[B11] Douglas LM The bacillus of long life 1911 London: TC & EC Jack

[B12] Fleischmann RD Adams MD White O Clayton RA Kirkness EF Kerlavage AR Bult CJ Tomb JF Dougherty BA Merrick JM et al. Whole-genome random sequencing and assembly of *Haemophilus influenzae *Rd Science 1995 269 496 512 10.1126/science.7542800 7542800

[B13] Bolotin A Wincker P Mauger S Jaillon O Malarme K Weissenbach J Ehrlich SD Sorokin A The complete genome sequence of the lactic acid bacterium *Lactococcus lactis *ssp. *lactis *IL1403 Genome Res 2001 11 731 753 10.1101/gr.GR-1697R 11337471 PMC311110

[B14] Kleerebezem M Boekhorst J van Kranenburg R Molenaar D Kuipers OP Leer R Tarchini R Peters SA Sandbrink HM Fiers MWEJ Complete genome sequence of *Lactobacillus plantarum *WCFS1 Proc Natl Acad Sci USA 2003 100 1990 1995 10.1073/pnas.0337704100 12566566 PMC149946

[B15] Johnson BR Klaenhammer TR Impact of genomics on the field of probiotic research: historical perspectives to modern paradigms Antonie Van Leeuwenhoek 2014 24748373 10.1007/s10482-014-0171-y PMC4064118

[B16] de Vos WM Systems solutions by lactic acid bacteria: from paradigms to practice Microb Cell Fact 2011 10 Suppl 1 S2 10.1186/1475-2859-10-S1-S2 21995776 PMC3231926

[B17] Linares DM Kok J Poolman B Genome sequences of *Lactococcus lactis *MG1363 (revised) and NZ9000 and comparative physiological studies J Bacteriol 2010 192 5806 5812 10.1128/JB.00533-10 20639323 PMC2953693

[B18] de Ruyter PG Kuipers OP de Vos WM Controlled gene expression systems for *Lactococcus lactis *with the food-grade inducer nisin Appl Environ Microbiol 1996 62 3662 3667 8837421 10.1128/aem.62.10.3662-3667.1996 PMC168174

[B19] Makarova K Slesarev A Wolf Y Sorokin A Mirkin B Koonin E Pavlov A Pavlova N Karamychev V Polouchine N Comparative genomics of the lactic acid bacteria Proc Natl Acad Sci USA 2006 103 15611 15616 10.1073/pnas.0607117103 17030793 PMC1622870

[B20] de Vos WM Vos P de Haard H Boerrigter I Cloning and expression of the *Lactococcus lactis *subsp. *cremoris *SK11 gene encoding an extracellular serine proteinase Gene 1989 85 169 176 10.1016/0378-1119(89)90477-0 2515994

[B21] Ainsworth S Zomer A de Jager V Bottacini F van Hijum SA Mahony J van Sinderen D Complete genome of *Lactococcus lactis *subsp. *cremoris *UC509.9, host for a model lactococcal P335 bacteriophage Genome Announc 2013 1 23405300 10.1128/genomeA.00119-12 PMC3569286

[B22] Kelly WJ Altermann E Lambie SC Leahy SC Interaction between the genomes of *Lactococcus lactis *and phages of the P335 species Front Microbiol 2013 4 257 24009606 10.3389/fmicb.2013.00257 PMC3757294

[B23] Siezen RJ Bayjanov J Renckens B Wels M van Hijum SAFT Molenaar D van Hylckama Vlieg JET Complete genome sequence of *Lactococcus lactis *subsp. *lactis *KF147, a plant-associated lactic acid bacterium J Bacteriol 2010 192 2649 2650 10.1128/JB.00276-10 20348266 PMC2863553

[B24] Settanni L Moschetti G Non-starter lactic acid bacteria used to improve cheese quality and provide health benefits Food Microbiol 2010 27 691 697 10.1016/j.fm.2010.05.023 20630311

[B25] Papanikolaou Z Hatzikamari M Georgakopoulos P Yiangou M Litopoulou-Tzanetaki E Tzanetakis N Selection of dominant NSLAB from a mature traditional cheese according to their technological properties and *in vitro *intestinal challenges J Food Sci 2012 77 M298 306 10.1111/j.1750-3841.2012.02685.x 23163947

[B26] Broadbent JR Hughes JE Welker DL Tompkins TA Steele JL Complete genome sequence for *Lactobacillus helveticus *CNRZ 32, an industrial cheese starter and cheese flavor adjunct Genome Announc 2013 1 23969047 10.1128/genomeA.00590-13 PMC3751602

[B27] Luesink EJ van Herpen REMA Grossiord BP Kuipers OP de Vos WM Transcriptional activation of the glycolytic las operon and catabolite repression of the gal operon in Lactococcus lactis are mediated by the catabolite control protein CcpA Mol Microbiol 1998 30 789 798 10.1046/j.1365-2958.1998.01111.x 10094627

[B28] Zomer AL Buist G Larsen R Kok J Kuipers OP Time-resolved determination of the CcpA regulon of *Lactococcus lactis *subsp. *cremoris *MG1363 J Bacteriol 2007 189 1366 1381 10.1128/JB.01013-06 17028270 PMC1797362

[B29] Loll B Saenger W Biesiadka J Structure of full-length transcription regulator CcpA in the apo form Biochim Biophys Acta 2007 1774 732 736 10.1016/j.bbapap.2007.03.020 17500051

[B30] Aleksandrzak-Piekarczyk T Polak J Jezierska B Renault P Bardowski J Genetic characterization of the CcpA-dependent, cellobiose-specific PTS system comprising CelB, PtcB and PtcA that transports lactose in *Lactococcus lactis *IL1403 Int J Food Microbiol 2011 145 186 194 10.1016/j.ijfoodmicro.2010.12.011 21262549

[B31] Barrangou R Azcarate-Peril MA Duong T Conners SB Kelly RM Klaenhammer TR Global analysis of carbohydrate utilization by *Lactobacillus acidophilus *using cDNA microarrays Proc Natl Acad Sci USA 2006 103 3816 3821 10.1073/pnas.0511287103 16505367 PMC1533782

[B32] Andersen JM Barrangou R Hachem MA Lahtinen SJ Goh YJ Svensson B Klaenhammer TR Transcriptional analysis of prebiotic uptake and catabolism by *Lactobacillus acidophilus *NCFM PLoS One 2012 7 e44409 10.1371/journal.pone.0044409 23028535 PMC3446993

[B33] McLeod A Snipen L Naterstad K Axelsson L Global transcriptome response in *Lactobacillus sakei *during growth on ribose BMC Microbiol 2011 11 145 10.1186/1471-2180-11-145 21702908 PMC3146418

[B34] Mazzeo MF Cacace G Peluso A Zotta T Muscariello L Vastano V Parente E Siciliano RA Effect of inactivation of ccpA and aerobic growth in *Lactobacillus plantarum*: A proteomic perspective J Proteomics 2012 75 4050 4061 10.1016/j.jprot.2012.05.019 22634038

[B35] Muscariello L Marino C Capri U Vastano V Marasco R Sacco M CcpA and three newly identified proteins are involved in biofilm development in *Lactobacillus plantarum* J Basic Microbiol 2013 53 62 71 10.1002/jobm.201100456 22585750

[B36] van den Bogaard PTC Kleerebezem M Kuipers OP de Vos WM Control of lactose transport, beta-galactosidase activity, and glycolysis by CcpA in *Streptococcus thermophilus*: evidence for carbon catabolite repression by a non-phosphoenolpyruvate-dependent phosphotransferase system sugar J Bacteriol 2000 182 5982 5989 10.1128/JB.182.21.5982-5989.2000 11029416 PMC94730

[B37] Gao P Pinkston KL Bourgogne A Cruz MR Garsin DA Murray BE Harvey BR Library screen identifies *Enterococcus faecalis *CcpA, the catabolite control protein A, as an effector of Ace, a collagen adhesion protein linked to virulence J Bacteriol 2013 195 4761 4768 10.1128/JB.00706-13 23974022 PMC3807442

[B38] Carvalho AL Turner DL Fonseca LL Solopova A Catarino T Kuipers OP Voit EO Neves AR Santos H Metabolic and transcriptional analysis of acid stress in *Lactococcus lactis*, with a focus on the kinetics of lactic acid pools PLoS One 2013 8 e68470 10.1371/journal.pone.0068470 23844205 PMC3700934

[B39] de Jong A Hansen ME Kuipers OP Kilstrup M Kok J The transcriptional and gene regulatory network of *Lactococcus lactis *MG1363 during growth in milk PLoS ONE 2013 8 e53085 10.1371/journal.pone.0053085 23349698 PMC3547956

[B40] Groot Kormelink T Koenders E Hagemeijer Y Overmars L Siezen RJ de Vos WM Francke C Comparative genome analysis of central nitrogen metabolism and its control by GlnR in the class Bacilli BMC Genomics 2012 13 191 10.1186/1471-2164-13-191 22607086 PMC3412718

[B41] Larsen R Kloosterman TG Kok J Kuipers OP GlnR-mediated regulation of nitrogen metabolism in *Lactococcus lactis* J Bacteriol 2006 188 4978 4982 10.1128/JB.00025-06 16788206 PMC1483007

[B42] den Hengst CD van Hijum SAFT Geurts JMW Nauta A Kok J Kuipers OP The *Lactococcus lactis *CodY regulon: identification of a conserved cis-regulatory element J Biol Chem 2005 280 34332 34342 10.1074/jbc.M502349200 16040604

[B43] Guedon E Sperandio B Pons N Ehrlich SD Renault P Overall control of nitrogen metabolism in *Lactococcus lactis *by CodY, and possible models for CodY regulation in Firmicutes Microbiology 2005 151 3895 3909 10.1099/mic.0.28186-0 16339935

[B44] Marugg JD van Kranenburg R Laverman P Rutten GA de Vos WM Identical transcriptional control of the divergently transcribed prtP and prtM genes that are required for proteinase production in *Lactococcus lactis *SK11 J Bacteriol 1996 178 1525 1531 8626277 10.1128/jb.178.6.1525-1531.1996 PMC177834

[B45] Dressaire C Redon E Gitton C Loubiere P Monnet V Cocaign-Bousquet M Investigation of the adaptation of *Lactococcus lactis *to isoleucine starvation integrating dynamic transcriptome and proteome information Microb Cell Fact 2011 10 Suppl 1 S18 10.1186/1475-2859-10-S1-S18 21995707 PMC3236307

[B46] Liu F Du L Du P Huo G Possible promoter regions within the proteolytic system in *Streptococcus thermophilus *and their interaction with the CodY homolog FEMS Microbiol Lett 2009 297 164 172 10.1111/j.1574-6968.2009.01672.x 19552712

[B47] Hendriksen WT Bootsma HJ Estevao S Hoogenboezem T de Jong A de Groot R Kuipers OP Hermans PWM CodY of *Streptococcus pneumoniae*: link between nutritional gene regulation and colonization J Bacteriol 2008 190 590 601 10.1128/JB.00917-07 18024519 PMC2223708

[B48] McDowell EJ Callegari EA Malke H Chaussee MS CodY-mediated regulation of *Streptococcus pyogenes *exoproteins BMC Microbiol 2012 12 114 10.1186/1471-2180-12-114 22721528 PMC3438106

[B49] Branco dos Santos F de Vos WM Teusink B Towards metagenome-scale models for industrial applications - the case of Lactic Acid Bacteria Curr Opin Biotechnol 2013 24 200 206 10.1016/j.copbio.2012.11.003 23200025

[B50] Bachmann H de Wilt L Kleerebezem M van Hylckama Vlieg JE Time-resolved genetic responses of *Lactococcus lactis *to a dairy environment Environ Microbiol 2010 12 1260 1270 10.1111/j.1462-2920.2010.02168.x 20192965

[B51] Cretenet M Laroute V Ulve V Jeanson S Nouaille S Even S Piot M Girbal L Le Loir Y Loubiere P Dynamic analysis of the *Lactococcus lactis *transcriptome in cheeses made from milk concentrated by ultrafiltration reveals multiple strategies of adaptation to stresses Appl Environ Microbiol 2011 77 247 257 10.1128/AEM.01174-10 21075879 PMC3019719

[B52] Le Boucher C Courant F Jeanson S Chereau S Maillard MB Royer AL Thierry A Dervilly-Pinel G Le Bizec B Lortal S First mass spectrometry metabolic fingerprinting of bacterial metabolism in a model cheese Food Chem 2013 141 1032 1040 10.1016/j.foodchem.2013.03.094 23790883

[B53] Taibi A Dabour N Lamoureux M Roy D LaPointe G Comparative transcriptome analysis of *Lactococcus lactis *subsp. *cremoris *strains under conditions simulating Cheddar cheese manufacture Int J Food Microbiol 2011 146 263 275 10.1016/j.ijfoodmicro.2011.02.034 21435733

[B54] Erkus O de Jager VCL Spus M van Alen-Boerrigter IJ van Rijswijck IMH Hazelwood L Janssen PWM van Hijum SAFT Kleerebezem M Smid EJ Multifactorial diversity sustains microbial community stability ISME J 2013 7 2126 2136 10.1038/ismej.2013.108 23823494 PMC3806261

[B55] Johansen P Vindelov J Arneborg N Brockmann E Development of quantitative PCR and metagenomics-based approaches for strain quantification of a defined mixed-strain starter culture Syst Appl Microbiol 2014 37 186 193 10.1016/j.syapm.2013.12.006 24582508

[B56] Aguado-Urda M Gibello A Blanco Mdel M Fernández-Garayzábal JF López-Alonso V López-Campos GH Global transcriptome analysis of *Lactococcus garvieae *strains in response to temperature PLoS One 2013 8 e79692 10.1371/journal.pone.0079692 24223997 PMC3817100

[B57] Brooijmans R Smit B Santos F van Riel J de Vos WM Hugenholtz J Heme and menaquinone induced electron transport in lactic acid bacteria Microb Cell Fact 2009 8 28 10.1186/1475-2859-8-28 19480672 PMC2696406

[B58] Jääskeläinen E Johansson P Kostiainen O Nieminen T Schmidt G Somervuo P Mohsina M Vanninen P Auvinen P Björkroth J Significance of heme-based respiration in meat spoilage caused by *Leuconostoc gasicomitatum* Appl Environ Microbiol 2013 79 1078 1085 10.1128/AEM.02943-12 23204416 PMC3568588

[B59] Pedersen MB Gaudu P Lechardeur D Petit MA Gruss A Aerobic respiration metabolism in lactic acid bacteria and uses in biotechnology Annu Rev Food Sci Technol 2012 3 37 58 10.1146/annurev-food-022811-101255 22385163

[B60] Zhai Z Douillard FP An H Wang G Guo X Luo Y Hao Y Proteomic characterization of the acid tolerance response in *Lactobacillus delbrueckii *subsp. *bulgaricus *CAUH1 and functional identification of a novel acid stress-related transcriptional regulator Ldb0677 Environ Microbiol 2013 16 1524 1537 24131507 10.1111/1462-2920.12280

[B61] Wunsche A Hammer E Bartholomae M Volker U Burkovski A Seidel G Hillen W CcpA forms complexes with CodY and RpoA in *Bacillus subtilis* FEBS J 2012 279 2201 2214 10.1111/j.1742-4658.2012.08604.x 22512862

[B62] Levdikov VM Blagova E Joseph P Sonenshein AL Wilkinson AJ The structure of CodY, a GTP- and isoleucine-responsive regulator of stationary phase and virulence in gram-positive bacteria J Biol Chem 2006 281 11366 11373 10.1074/jbc.M513015200 16488888

[B63] Geiger T Wolz C Intersection of the stringent response and the CodY regulon in low GC Gram-positive bacteria Int J Med Microbiol 2014 304 150 155 10.1016/j.ijmm.2013.11.013 24462007

[B64] Wolz C Geiger T Goerke C The synthesis and function of the alarmone (p)ppGpp in firmicutes Int J Med Microbiol 2010 300 142 147 10.1016/j.ijmm.2009.08.017 19783475

[B65] Winstedt L Frankenberg L Hederstedt L von Wachenfeldt C *Enterococcus faecalis *V583 contains a cytochrome bd-type respiratory oxidase J Bacteriol 2000 182 3863 3866 10.1128/JB.182.13.3863-3866.2000 10851008 PMC94564

[B66] Portela CAF Smart KF Tumanov S Cook GM Villas-Boas SG The global metabolic response of *Enterococcus faecalis *to oxygen J Bacteriol 2014 24659768 10.1128/JB.01354-13 PMC4010974

[B67] Brooijmans RJW Poolman B Schuurman-Wolters GK de Vos WM Hugenholtz J Generation of a membrane potential by Lactococcus lactis through aerobic electron transport J Bacteriol 2007 189 5203 5209 10.1128/JB.00361-07 17496098 PMC1951855

[B68] Oxaran V Ledue-Clier F Dieye Y Herry JM Pechoux C Meylheuc T Briandet R Juillard V Piard JC Pilus biogenesis in Lactococcus lactis: molecular characterization and role in aggregation and biofilm formation PLoS One 2012 7 e50989 10.1371/journal.pone.0050989 23236417 PMC3516528

[B69] Kankainen M Paulin L Tynkkynen S von Ossowski I Reunanen J Partanen P Satokari R Vesterlund S Hendrickx APA Lebeer S Comparative genomic analysis of *Lactobacillus rhamnosus *GG reveals pili containing a human- mucus binding protein Proc Natl Acad Sci USA 2009 106 17193 17198 10.1073/pnas.0908876106 19805152 PMC2746127

[B70] Segers ME Lebeer S *Lactobacillus rhamnosus *GG - host interactions Microb Cell Fact 2014 in press 10.1186/1475-2859-13-S1-S7 PMC4155824 25186587

[B71] Meyrand M Guillot A Goin M Furlan S Armalyte J Kulakauskas S Cortes-Perez NG Thomas G Chat S Pechoux C Surface proteome analysis of a natural isolate of *Lactococcus lactis *reveals the presence of pili able to bind human intestinal epithelial cells Mol Cell Proteomics 2013 12 3935 3947 10.1074/mcp.M113.029066 24002364 PMC3861735

[B72] Vaughan EE de Vries MC Zoetendal EG Ben-Amor K Akkermans ADL de Vos WM The intestinal LABs Antonie Van Leeuwenhoek 2002 82 341 352 10.1023/A:1020672724450 12369201

[B73] Walter J Ecological role of lactobacilli in the gastrointestinal tract: implications for fundamental and biomedical research Appl Environ Microbiol 2008 74 4985 4996 10.1128/AEM.00753-08 18539818 PMC2519286

[B74] Bello FD Walter J Hammes WP Hertel C Increased complexity of the species composition of lactic acid bacteria in human feces revealed by alternative incubation condition Microb Ecol 2003 45 455 463 10.1007/s00248-003-2001-z 12704557

[B75] Heilig HGHJ Zoetendal EG Vaughan EE Marteau P Akkermans ADL de Vos WM Molecular diversity of Lactobacillus spp. and other lactic acid bacteria in the human intestine as determined by specific amplification of 16S ribosomal DNA Appl Environ Microbiol 2002 68 114 123 10.1128/AEM.68.1.114-123.2002 11772617 PMC126540

[B76] Ahrne S Nobaek S Jeppsson B Adlerberth I Wold AE Molin G The normal *Lactobacillus *flora of healthy human rectal and oral mucosa J Appl Microbiol 1998 85 88 94 10.1046/j.1365-2672.1998.00480.x 9721659

[B77] Molin G Jeppsson B Johansson ML Ahrné S Nobaek S Stahl M Bengmark S Numerical taxonomy of *Lactobacillus *spp. associated with healthy and diseased mucosa of the human intestines J Appl Bacteriol 1993 74 314 323 10.1111/j.1365-2672.1993.tb03031.x 8468264

[B78] Klijn N Weerkamp AH de Vos WM Genetic marking of *Lactococcus lactis *shows its survival in the human gastrointestinal tract Appl Environ Microbiol 1995 61 2771 2774 7618890 10.1128/aem.61.7.2771-2774.1995 PMC167550

[B79] David LA Maurice CF Carmody RN Gootenberg DB Button JE Wolfe BE Ling AV Devlin AS Varma Y Fischbach MA Diet rapidly and reproducibly alters the human gut microbiome Nature 2014 505 559 563 24336217 10.1038/nature12820 PMC3957428

[B80] Douillard FP Ribbera A Kant R Pietila TE Jarvinen HM Messing M Randazzo CL Paulin L Laine P Ritari J Comparative genomic and functional analysis of 100 *Lactobacillus rhamnosus *strains and their comparison with strain GG PLoS Genet 2013 9 e1003683 10.1371/journal.pgen.1003683 23966868 PMC3744422

[B81] Macklaim J Fernandes A Di Bella J Hammond J-A Reid G Gloor G Comparative meta-RNA-seq of the vaginal microbiota and differential expression by *Lactobacillus iners *in health and dysbiosis Microbiome 2013 1 12 10.1186/2049-2618-1-12 24450540 PMC3971606

[B82] de Vos WM de Vos EAJ Role of the intestinal microbiome in health and disease: from correlation to causation Nutr Rev 2012 70 Suppl 1 S45 56 22861807 10.1111/j.1753-4887.2012.00505.x

[B83] Karlsson FH Tremaroli V Nookaew I Bergstrom G Behre CJ Fagerberg B Nielsen J Backhed F Gut metagenome in European women with normal, impaired and diabetic glucose control Nature 2013 498 99 103 10.1038/nature12198 23719380

[B84] de Vos WM Nieuwdorp M Genomics: a gut prediction Nature 2013 498 48 49 10.1038/nature12251 23719383

[B85] Benítez-Páez A Belda-Ferre P Simón-Soro A Mira A Microbiota diversity and gene expression dynamics in human oral biofilms BMC Genomics 2014 15 311 10.1186/1471-2164-15-311 24767457 PMC4234424

[B86] Hojo K Mizoguchi C Taketomo N Ohshima T Gomi K Arai T Maeda N Distribution of salivary *Lactobacillus *and *Bifidobacterium *species in periodontal health and disease Biosci Biotechnol Biochem 2007 71 152 157 10.1271/bbb.60420 17213656

[B87] Strahinic I Busarcevic M Pavlica D Milasin J Golic N Topisirovic L Molecular and biochemical characterizations of human oral lactobacilli as putative probiotic candidates Oral Microbiol Immunol 2007 22 111 117 10.1111/j.1399-302X.2007.00331.x 17311634

[B88] Jagtap P McGowan T Bandhakavi S Tu ZJ Seymour S Griffin TJ Rudney JD Deep metaproteomic analysis of human salivary supernatant Proteomics 2012 12 992 1001 10.1002/pmic.201100503 22522805 PMC3517020

[B89] Ahola AJ Yli-Knuuttila H Suomalainen T Poussa T Ahlstrom A Meurman JH Korpela R Short-term consumption of probiotic-containing cheese and its effect on dental caries risk factors Arch Oral Biol 2002 47 799 804 10.1016/S0003-9969(02)00112-7 12446187

[B90] Nadkarni MA Chen Z Wilkins MR Hunter N Comparative genome analysis of *Lactobacillus rhamnosus *clinical isolates from initial stages of dental pulp infection: identification of a new exopolysaccharide cluster PLoS ONE 2014 9 e90643 10.1371/journal.pone.0090643 24632842 PMC3954586

[B91] Qin J Li R Raes J Arumugam M Burgdorf KS Manichanh C Nielsen T Pons N Levenez F Yamada T A human gut microbial gene catalogue established by metagenomic sequencing Nature 2010 464 59 65 10.1038/nature08821 20203603 PMC3779803

[B92] Louis P Scott KP Duncan SH Flint HJ Understanding the effects of diet on bacterial metabolism in the large intestine J Appl Microbiol 2007 102 1197 1208 10.1111/j.1365-2672.2007.03322.x 17448155

[B93] Harmsen HJM Raangs GC He T Degener JE Welling GW Extensive set of 16S rRNA-based probes for detection of bacteria in human feces Appl Environ Microbiol 2002 68 2982 2990 10.1128/AEM.68.6.2982-2990.2002 12039758 PMC123985

[B94] Booijink CCGM El-Aidy S Rajilić-Stojanović M Heilig HGHJ Troost FJ Smidt H Kleerebezem M De Vos WM Zoetendal EG High temporal and inter-individual variation detected in the human ileal microbiota Environ Microbiol 2010 12 3213 3227 10.1111/j.1462-2920.2010.02294.x 20626454

[B95] Zoetendal EG Raes J van den Bogert B Arumugam M Booijink CCGM Troost FJ Bork P Wels M de Vos WM Kleerebezem M The human small intestinal microbiota is driven by rapid uptake and conversion of simple carbohydrates ISME J 2012 6 1415 1426 10.1038/ismej.2011.212 22258098 PMC3379644

[B96] van den Bogert B Erkus O Boekhorst J de Goffau M Smid EJ Zoetendal EG Kleerebezem M Diversity of human small intestinal *Streptococcus *and *Veillonella *populations FEMS Microbiol Ecol 2013 85 376 388 10.1111/1574-6941.12127 23614882

[B97] Bernardeau M Guguen M Vernoux JP Beneficial lactobacilli in food and feed: long-term use, biodiversity and proposals for specific and realistic safety assessments FEMS Microbiol Rev 2006 30 487 513 10.1111/j.1574-6976.2006.00020.x 16774584

[B98] Vesa T Pochart P Marteau P Pharmacokinetics of *Lactobacillus plantarum *NCIMB 8826, *Lactobacillus fermentum *KLD, and *Lactococcus lactis *MG 1363 in the human gastrointestinal tract Aliment Pharmacol Ther 2000 14 823 828 10.1046/j.1365-2036.2000.00763.x 10848668

[B99] Lahti L Salonen A Kekkonen RA Salojarvi J Jalanka-Tuovinen J Palva A Oresic M de Vos WM Associations between the human intestinal microbiota, *Lactobacillus rhamnosus *GG and serum lipids indicated by integrated analysis of high-throughput profiling data PeerJ 2013 26 23638368 10.7717/peerj.32 PMC3628737

[B100] Selle K Klaenhammer TR Genomic and phenotypic evidence for probiotic influences of *Lactobacillus gasseri *on human health FEMS Microbiol Rev 2013 37 915 935 23488471 10.1111/1574-6976.12021

[B101] Denou E Pridmore RD Berger B Panoff JM Arigoni F Brussow H Identification of genes associated with the long-gut-persistence phenotype of the probiotic *Lactobacillus johnsonii *strain NCC533 using a combination of genomics and transcriptome analysis J Bacteriol 2008 190 3161 3168 10.1128/JB.01637-07 18223069 PMC2347406

[B102] Toh H Oshima K Nakano A Takahata M Murakami M Takaki T Nishiyama H Igimi S Hattori M Morita H Genomic Adaptation of the *Lactobacillus casei *Group PLoS ONE 2013 8 e75073 10.1371/journal.pone.0075073 24116025 PMC3792948

[B103] Cai H Rodríguez BT Zhang W Broadbent JR Steele JL Genotypic and phenotypic characterization of *Lactobacillus casei *strains isolated from different ecological niches suggests frequent recombination and niche specificity Microbiol 2007 153 2655 2665 10.1099/mic.0.2007/006452-0 17660430

[B104] Raftis EJ Salvetti E Torriani S Felis GE O'Toole PW Genomic diversity of *Lactobacillus salivarius* Appl Environ Microbiol 2011 77 954 965 10.1128/AEM.01687-10 21131523 PMC3028724

[B105] Corr SC Li Y Riedel CU O'Toole PW Hill C Gahan CG Bacteriocin production as a mechanism for the antiinfective activity of *Lactobacillus salivarius *UCC118 Proc Natl Acad Sci USA 2007 104 7617 7621 10.1073/pnas.0700440104 17456596 PMC1863472

[B106] Claesson MJ Li Y Leahy S Canchaya C van Pijkeren JP Cerdeño-Tárraga AM Parkhill J Flynn S O'Sullivan GC Collins JK Multireplicon genome architecture of *Lactobacillus salivarius* Proc Natl Acad Sci USA 2006 103 6718 6723 10.1073/pnas.0511060103 16617113 PMC1436024

[B107] Naito S Hayashidani H Kaneko K Ogawa M Benno Y Development of intestinal lactobacilli in normal piglets J Appl Bacteriol 1995 79 230 236 10.1111/j.1365-2672.1995.tb00940.x 7592119

[B108] Molin G Johansson ML Ståhl M Ahrné S Andersson R Jeppsson B Bengmark S Systematics of the *Lactobacillus *population on rat intestinal mucosa with special reference to *Lactobacillus reuteri* Antonie Van Leeuwenhoek 1992 61 175 183 10.1007/BF00584224 1325752

[B109] Frese SA Benson AK Tannock GW Loach DM Kim J Zhang M Oh PL Heng NCK Patil PB Juge N The Evolution of host specialization in the vertebrate gut symbiont *Lactobacillus reuteri* PLoS Genet 2011 7 e1001314 10.1371/journal.pgen.1001314 21379339 PMC3040671

[B110] Moran NA Plague GR Genomic changes following host restriction in bacteria Curr Opin Genet Dev 2004 14 627 633 10.1016/j.gde.2004.09.003 15531157

[B111] Forde BM Neville BA O'Donnell MM Riboulet-Bisson E Claesson MJ Coghlan A Ross RP O'Toole PW Genome sequences and comparative genomics of two *Lactobacillus ruminis *strains from the bovine and human intestinal tracts Microb Cell Fact 2011 10 Suppl 1 S13 10.1186/1475-2859-10-S1-S13 21995554 PMC3231920

[B112] Altermann E Russell WM Azcarate-Peril MA Barrangou R Buck BL McAuliffe O Souther N Dobson A Duong T Callanan M Complete genome sequence of the probiotic lactic acid bacterium *Lactobacillus acidophilus *NCFM Proc Natl Acad Sci USA 2005 102 3906 3912 10.1073/pnas.0409188102 15671160 PMC554803

[B113] Callanan M Kaleta P O'Callaghan J O'Sullivan O Jordan K McAuliffe O Sangrador-Vegas A Slattery L Fitzgerald GF Beresford T Ross RP Genome sequence of *Lactobacillus helveticus*, an organism distinguished by selective gene loss and insertion sequence element expansion J Bacteriol 2008 190 727 735 10.1128/JB.01295-07 17993529 PMC2223680

[B114] Douillard FP Ribbera A Järvinen HM Kant R Pietilä TE Randazzo C Paulin L Laine PK Caggia C von Ossowski I Comparative genomic and functional analysis of *Lactobacillus casei *and *Lactobacillus rhamnosus *strains marketed as probiotics Appl Environ Microbiol 2013 79 1923 1933 10.1128/AEM.03467-12 23315726 PMC3592221

[B115] van Pijkeren JP Canchaya C Ryan KA Li Y Claesson MJ Sheil B Steidler L O'Mahony L Fitzgerald GF van Sinderen D O'Toole PW Comparative and functional analysis of sortase-dependent proteins in the predicted secretome of *Lactobacillus salivarius *UCC118 Appl Environ Microbiol 2006 72 4143 4153 10.1128/AEM.03023-05 16751526 PMC1489637

[B116] Call EK Klaenhammer TR Relevance and application of sortase and sortase-dependent proteins in lactic acid bacteria Front Microbiol 2013 4 73 23579319 10.3389/fmicb.2013.00073 PMC3619620

[B117] Neville BA Forde BM Claesson MJ Darby T Coghlan A Nally K Ross RP O'Toole PW Characterization of pro-inflammatory flagellin proteins produced by *Lactobacillus ruminis *and related motile lactobacilli PLoS ONE 2012 7 e40592 10.1371/journal.pone.0040592 22808200 PMC3393694

[B118] Azcarate-Peril MA Altermann E Goh YJ Tallon R Sanozky-Dawes RB Pfeiler EA O'Flaherty S Buck BL Dobson A Duong T Analysis of the genome sequence of *Lactobacillus gasseri *ATCC 33323 reveals the molecular basis of an autochthonous intestinal organism Appl Environ Microbiol 2008 74 4610 4625 10.1128/AEM.00054-08 18539810 PMC2519322

[B119] Yanagihara S Hirota T Yamamoto N Transcriptional response of *Lactobacillus acidophilus *L-92 after attachment to epithelial Caco-2 cells J Biosci Bioeng 2012 114 582 585 10.1016/j.jbiosc.2012.07.001 22841868

[B120] Ashida N Yanagihara S Shinoda T Yamamoto N Characterization of adhesive molecule with affinity to Caco-2 cells in *Lactobacillus acidophilus *by proteome analysis J Biosci Bioeng 2011 112 333 337 10.1016/j.jbiosc.2011.06.001 21763196

[B121] Konstantinov SR Smidt H de Vos WM Bruijns SCM Singh SK Valence F Molle D Lortal S Altermann E Klaenhammer TR van Kooyk Y S layer protein A of *Lactobacillus acidophilus *NCFM regulates immature dendritic cell and T cell functions Proc Natl Acad Sci USA 2008 105 19474 19479 10.1073/pnas.0810305105 19047644 PMC2592362

[B122] Johnson B Selle K O'Flaherty S Goh YJ Klaenhammer T Identification of extracellular surface-layer associated proteins in *Lactobacillus acidophilus *NCFM Microbiology 2013 159 2269 2282 10.1099/mic.0.070755-0 24002751 PMC3836491

[B123] Koskenniemi K Laakso K Koponen J Kankainen M Greco D Auvinen P Savijoki K Nyman TA Surakka A Salusjärvi T Proteomics and transcriptomics characterization of bile stress response in probiotic *Lactobacillus rhamnosus *GG Mol Cell Prot 2011 10 21078892 10.1074/mcp.M110.002741 PMC3033674

[B124] Koponen J Laakso K Koskenniemi K Kankainen M Savijoki K Nyman TA de Vos WM Tynkkynen S Kalkkinen N Varmanen P Effect of acid stress on protein expression and phosphorylation in *Lactobacillus rhamnosus *GG J Proteomics 2012 75 1357 1374 10.1016/j.jprot.2011.11.009 22119544

[B125] Alcántara C Zúñiga M Proteomic and transcriptomic analysis of the response to bile stress of *Lactobacillus casei *BL23 Microbiology 2012 158 1206 1218 10.1099/mic.0.055657-0 22322960

[B126] Goh YJ Klaenhammer TR A functional glycogen biosynthesis pathway in *Lactobacillus acidophilus*: expression and analysis of the *glg *operon Mol Microbiol 2013 89 1187 1200 10.1111/mmi.12338 23879596 PMC4282360

[B127] Bron PA Grangette C Mercenier A de Vos WM Kleerebezem M Identification of *Lactobacillus plantarum *genes that are induced in the gastrointestinal tract of mice J Bacteriol 2004 186 5721 5729 10.1128/JB.186.17.5721-5729.2004 15317777 PMC516819

[B128] Troost FJ van Baarlen P Lindsey P Kodde A de Vos WM Kleerebezem M Brummer RJ Identification of the transcriptional response of human intestinal mucosa to *Lactobacillus plantarum *WCFS1 *in vivo* BMC Genomics 2008 9 374 10.1186/1471-2164-9-374 18681965 PMC2519092

[B129] van Baarlen P Troost FJ van Hemert S van der Meer C de Vos WM de Groot PJ Hooiveld GJEJ Brummer RJ Kleerebezem M Differential NF-kappaB pathways induction by *Lactobacillus plantarum *in the duodenum of healthy humans correlating with immune tolerance Proc Natl Acad Sci USA 2009 106 2371 2376 10.1073/pnas.0809919106 19190178 PMC2650163

[B130] Marco ML Peters THF Bongers RS Molenaar D Van Hemert S Sonnenburg JL Gordon JI Kleerebezem M Lifestyle of *Lactobacillus plantarum *in the mouse caecum Environ Microbiol 2009 11 2747 2757 10.1111/j.1462-2920.2009.02001.x 19638173 PMC2978903

[B131] Marco ML de Vries MC Wels M Molenaar D Mangell P Ahrne S de Vos WM Vaughan EE Kleerebezem M Convergence in probiotic *Lactobacillus *gut-adaptive responses in humans and mice ISME J 2010 4 1481 1484 10.1038/ismej.2010.61 20505752

[B132] Reverón I de las Rivas B Muñoz R López de Felipe F Genome-wide transcriptomic responses of a human isolate of *Lactobacillus plantarum *exposed to p-coumaric acid stress Molecular Nutrition & Food Research 2012 56 1848 1859 10.1002/mnfr.201200384 23065750

[B133] Bron PA Marco M Hoffer SM Van Mullekom E de Vos WM Kleerebezem M Genetic characterization of the bile salt response in *Lactobacillus plantarum *and analysis of responsive promoters *in vitro *and *in situ *in the gastrointestinal tract J Bacteriol 2004 186 7829 7835 10.1128/JB.186.23.7829-7835.2004 15547253 PMC529069

[B134] Hamon E Horvatovich P Izquierdo E Bringel F Marchioni E Aoude-Werner D Ennahar S Comparative proteomic analysis of *Lactobacillus plantarum *for the identification of key proteins in bile tolerance BMC Microbiol 2011 11 63 10.1186/1471-2180-11-63 21447177 PMC3073879

[B135] van Bokhorst-van de Veen H Smelt MJ Wels M van Hijum SAFT de Vos P Kleerebezem M Bron PA Genotypic adaptations associated with prolonged persistence of *Lactobacillus plantarum *in the murine digestive tract Biotechnol J 2013 8 895 904 10.1002/biot.201200259 24066356

[B136] Kim JF Jeong H Lee JS Choi SH Ha M Hur CG Kim JS Lee S Park HS Park YH Oh TK Complete genome sequence of *Leuconostoc citreum *KM20 J Bacteriol 2008 190 3093 3094 10.1128/JB.01862-07 18281406 PMC2293239

[B137] Lee K Pi K Effect of transient acid stress on the proteome of intestinal probiotic *Lactobacillus reuteri* Biochemistry (Mosc) 2010 75 460 465 10.1134/S0006297910040097 20618135

[B138] Denou E Berger B Barretto C Panoff JM Arigoni F Brussow H Gene expression of commensal *Lactobacillus johnsonii *strain NCC533 during in vitro growth and in the murine gut J Bacteriol 2007 189 8109 8119 10.1128/JB.00991-07 17827285 PMC2168692

[B139] Pendharkar S Magopane T Larsson PG de Bruyn G Gray GE Hammarstrom L Marcotte H Identification and characterisation of vaginal lactobacilli from South African women BMC Infect Dis 2013 13 43 10.1186/1471-2334-13-43 23351177 PMC3600991

[B140] Martínez-Peña MD Castro-Escarpulli G Aguilera-Arreola MG *Lactobacillus *species isolated from vaginal secretions of healthy and bacterial vaginosis-intermediate Mexican women: a prospective study BMC Infect Dis 2013 13 189 10.1186/1471-2334-13-189 23617246 PMC3655868

[B141] Zhou X Hansmann MA Davis CC Suzuki H Brown CJ Schutte U Pierson JD Forney LJ The vaginal bacterial communities of Japanese women resemble those of women in other racial groups FEMS Immunol Med Microbiol 2010 58 169 181 10.1111/j.1574-695X.2009.00618.x 19912342 PMC2868947

[B142] Ravel J Gajer P Abdo Z Schneider GM Koenig SSK McCulle SL Karlebach S Gorle R Russell J Tacket CO Vaginal microbiome of reproductive-age women Proc Natl Acad Sci USA 2011 108 Suppl 1 4680 4687 20534435 10.1073/pnas.1002611107 PMC3063603

[B143] Zhou X Brown CJ Abdo Z Davis CC Hansmann MA Joyce P Foster JA Forney LJ Differences in the composition of vaginal microbial communities found in healthy Caucasian and black women ISME J 2007 1 121 133 10.1038/ismej.2007.12 18043622

[B144] Drell T Lillsaar T Tummeleht L Simm J Aaspõllu A Vain E Saarma I Salumets A Donders GGG Metsis M Characterization of the vaginal micro- and mycobiome in asymptomatic reproductive-age Estonian women PLoS One 2013 8 e54379 10.1371/journal.pone.0054379 23372716 PMC3553157

[B145] Motevaseli E Shirzad M Raoofian R Hasheminasab SM Hatami M Dianatpour M Modarressi MH Differences in vaginal lactobacilli composition of Iranian healthy and bacterial vaginosis infected women: a comparative analysis of their cytotoxic effects with commercial vaginal probiotics Iran Red Crescent Med J 2013 15 199 206 10.5812/ircmj.3533 23983998 PMC3745747

[B146] Antonio MA Hawes SE Hillier SL The identification of vaginal *Lactobacillus *species and the demographic and microbiologic characteristics of women colonized by these species J Infect Dis 1999 180 1950 1956 10.1086/315109 10558952

[B147] Shipitsyna E Roos A Datcu R Hallén A Fredlund H Jensen JS Engstrand L Unemo M Composition of the vaginal microbiota in women of reproductive age--sensitive and specific molecular diagnosis of bacterial vaginosis is possible? PLoS One 2013 8 e60670 10.1371/journal.pone.0060670 23585843 PMC3621988

[B148] O'Hanlon DE Moench TR Cone RA Vaginal pH and microbicidal lactic acid when lactobacilli dominate the microbiota PLoS One 2013 8 e80074 10.1371/journal.pone.0080074 24223212 PMC3819307

[B149] Vera Pingitore E Hebert EM Nader-Macias ME Sesma F Characterization of salivaricin CRL 1328, a two-peptide bacteriocin produced by *Lactobacillus salivarius *CRL 1328 isolated from the human vagina Res Microbiol 2009 160 401 408 10.1016/j.resmic.2009.06.009 19591924

[B150] Kaewnopparat S Dangmanee N Kaewnopparat N Srichana T Chulasiri M Settharaksa S *In vitro *probiotic properties of *Lactobacillus fermentum *SK5 isolated from vagina of a healthy woman Anaerobe 2013 22 6 13 23624069 10.1016/j.anaerobe.2013.04.009

[B151] Homayouni A Bastani P Ziyadi S Mohammad-Alizadeh-Charandabi S Ghalibaf M Mortazavian AM Mehrabany EV Effects of probiotics on the recurrence of bacterial vaginosis: a review J Low Genit Tract Dis 2014 18 79 86 10.1097/LGT.0b013e31829156ec 24299970

[B152] Mastromarino P Vitali B Mosca L Bacterial vaginosis: a review on clinical trials with probiotics New Microbiol 2013 36 229 238 23912864

[B153] Mendes-Soares H Suzuki H Hickey RJ Forney LJ Comparative functional genomics of *Lactobacillus *spp. reveals possible mechanisms for specialization of vaginal lactobacilli to their environment J Bacteriol 2014 196 1458 1470 10.1128/JB.01439-13 24488312 PMC3993339

[B154] Macklaim JM Gloor GB Anukam KC Cribby S Reid G At the crossroads of vaginal health and disease, the genome sequence of *Lactobacillus iners *AB-1 Proc Natl Acad Sci USA 2011 108 Suppl 1 4688 4695 21059957 10.1073/pnas.1000086107 PMC3063587

[B155] Murata K Hoshina T Saito M Ohkusu K Yamamura K Tanoue Y Ihara K Hara T Bacterial pericarditis caused by *Lactobacillus iners *in an infant Diagn Microbiol Infect Dis 2012 74 181 182 10.1016/j.diagmicrobio.2012.06.008 22818098

[B156] Tanaka Y Watanabe J Mogi Y Monitoring of the microbial communities involved in the soy sauce manufacturing process by PCR-denaturing gradient gel electrophoresis Food Microbiol 2012 31 100 106 10.1016/j.fm.2012.02.005 22475947

[B157] McMillan A Macklaim JM Burton JP Reid G Adhesion of *Lactobacillus iners *AB-1 to human fibronectin: a key mediator for persistence in the vagina? Reprod Sci 2013 20 791 796 10.1177/1933719112466306 23202727

[B158] Osset J Bartolomé RM García E Andreu A Assessment of the capacity of *Lactobacillus *to inhibit the growth of uropathogens and block their adhesion to vaginal epithelial cells J Infect Dis 2001 183 485 491 10.1086/318070 11133381

[B159] Saunders S Bocking A Challis J Reid G Effect of *Lactobacillus *challenge on *Gardnerella vaginalis *biofilms Colloids Surf B Biointerfaces 2007 55 138 142 10.1016/j.colsurfb.2006.11.040 17234391

[B160] Rampersaud R Planet PJ Randis TM Kulkarni R Aguilar JL Lehrer RI Ratner AJ Inerolysin, a cholesterol-dependent cytolysin produced by *Lactobacillus iners* J Bacteriol 2011 193 1034 1041 10.1128/JB.00694-10 21169489 PMC3067590

[B161] Malik S Petrova MI Claes IJJ Verhoeven TLA Busschaert P Vaneechoutte M Lievens B Lambrichts I Siezen RJ Balzarini J The highly autoaggregative and adhesive phenotype of the vaginal *Lactobacillus plantarum *strain CMPG5300 is sortase dependent Appl Environ Microbiol 2013 79 4576 4585 10.1128/AEM.00926-13 23709503 PMC3719525

[B162] Bohbot JM Cardot JM Vaginal impact of the oral administration of total freeze-dried culture of LCR 35 in healthy women Infect Dis Obstet Gynecol 2012 2012 503648 22701297 10.1155/2012/503648 PMC3373062

[B163] Parma M Dindelli M Caputo L Redaelli A Quaranta L Candiani M The role of vaginal *Lactobacillus Rhamnosus *(Normogin(R)) in preventing Bacterial Vaginosis in women with history of recurrences, undergoing surgical menopause: a prospective pilot study Eur Rev Med Pharmacol Sci 2013 17 1399 1403 23740456

[B164] Colodner R Edelstein H Chazan B Raz R Vaginal colonization by orally administered *Lactobacillus rhamnosus *GG Isr Med Assoc J 2003 5 767 769 14650098

[B165] EFSA BIOHAZ Panel (EFSA Panel on Biological Hazards) Scientific Opinion on the maintenance of the list of QPS biological agents intentionally added to food and feed (2013 update) EFSA Journal 2013 11 108

[B166] Foulquié Moreno MR Sarantinopoulos P Tsakalidou E De Vuyst L The role and application of enterococci in food and health Int J Food Microbiol 2006 106 1 24 10.1016/j.ijfoodmicro.2005.06.026 16216368

[B167] Lebreton F Willems RJL Gilmore MS Gilmore MS, Clewell DB, Ike Y, Shankar N Enterococcus Diversity, Origins in Nature, and Gut Colonization Enterococci: From commensals to leading causes of drug resistant infection 2014 Boston 24649510

[B168] Gilmore MS Lebreton F van Schaik W Genomic transition of enterococci from gut commensals to leading causes of multidrug-resistant hospital infection in the antibiotic era Curr Opin Microbiol 2013 16 10 16 10.1016/j.mib.2013.01.006 23395351 PMC3649759

[B169] Galloway-Peña J Roh JH Latorre M Qin X Murray BE Genomic and SNP analyses demonstrate a distant separation of the hospital and community-associated clades of *Enterococcus faecium* PLoS One 2012 7 e30187 10.1371/journal.pone.0030187 22291916 PMC3266884

[B170] Lukjancenko O Ussery DW Wassenaar TM Comparative genomics of *Bifidobacterium, Lactobacillus *and related probiotic genera Microb Ecol 2012 63 651 673 10.1007/s00248-011-9948-y 22031452 PMC3324989

[B171] Gouriet F Million M Henri M Fournier PE Raoult D *Lactobacillus rhamnosus *bacteremia: an emerging clinical entity Eur J Clin Microbiol Infect Dis 2012 31 2469 2480 10.1007/s10096-012-1599-5 22544343

[B172] Salminen MK Tynkkynen S Rautelin H Saxelin M Vaara M Ruutu P Sarna S Valtonen V Järvinen A *Lactobacillus *bacteremia during a rapid increase in probiotic use of *Lactobacillus rhamnosus *GG in Finland Clin Infect Dis 2002 35 1155 1160 10.1086/342912 12410474

[B173] Plumed-Ferrer C Uusikyla K Korhonen J von Wright A Characterization of *Lactococcus lactis *isolates from bovine mastitis Vet Microbiol 2013 167 592 599 10.1016/j.vetmic.2013.09.011 24080351

[B174] Hadjisymeou S Loizou P Kothari P *Lactococcus lactis cremoris *infection: not rare anymore? BMJ Case Rep 2013 2013 23667218 10.1136/bcr-2012-008479 PMC3669795

[B175] Inoue M Saito A Kon H Uchida H Koyama S Haryu S Sasaki T Nishijima M Subdural empyema due to *Lactococcus lactis cremoris*: case report Neurol Med Chir (Tokyo) 2014 54 341 317 10.2176/nmc.cr2012-0440 24257498 PMC4533472

[B176] Santer M Joseph Lister: first use of a bacterium as a 'model organism' to illustrate the cause of infectious disease of humans Notes Rec R Soc Lond 2010 64 59 65 10.1098/rsnr.2009.0029 20503823

[B177] Zwielehner J Handschur M Michaelsen A Irez S Demel M Denner EBM Haslberger AG DGGE and real-time PCR analysis of lactic acid bacteria in bacterial communities of the phyllosphere of lettuce Mol Nutr Food Res 2008 52 614 623 10.1002/mnfr.200700158 18398868

[B178] Braem G De Vliegher S Verbist B Piessens V Van Coillie E De Vuyst L Leroy F Unraveling the microbiota of teat apices of clinically healthy lactating dairy cows, with special emphasis on coagulase-negative staphylococci J Dairy Sci 2013 96 1499 1510 10.3168/jds.2012-5493 23313004

[B179] Bayjanov JR Starrenburg MJC van der Sijde MR Siezen RJ van Hijum SAFT Genotype-phenotype matching analysis of 38 *Lactococcus lactis *strains using random forest methods BMC Microbiol 2013 13 68 10.1186/1471-2180-13-68 23530958 PMC3637802

[B180] McCutcheon JP Moran NA Extreme genome reduction in symbiotic bacteria Nat Rev Micro 2012 10 13 26 22064560 10.1038/nrmicro2670

[B181] Bolotin A Quinquis B Renault P Sorokin A Ehrlich SD Kulakauskas S Lapidus A Goltsman E Mazur M Pusch GD Complete sequence and comparative genome analysis of the dairy bacterium *Streptococcus thermophilus* Nat Biotechnol 2004 22 1554 1558 10.1038/nbt1034 15543133 PMC7416660

[B182] Pittet V Abegunde T Marfleet T Haakensen M Morrow K Jayaprakash T Schroeder K Trost B Byrns S Bergsveinson J Complete genome sequence of the beer spoilage organism *Pediococcus claussenii *ATCC BAA-344T J Bacteriol 2012 194 1271 1272 10.1128/JB.06759-11 22328764 PMC3294761

[B183] Broadbent JR Neeno-Eckwall EC Stahl B Tandee K Cai H Morovic W Horvath P Heidenreich J Perna NT Barrangou R Steele JL Analysis of the *Lactobacillus casei *supragenome and its influence in species evolution and lifestyle adaptation BMC Genomics 2012 13 533 10.1186/1471-2164-13-533 23035691 PMC3496567

[B184] Nicolas P Bessieres P Ehrlich SD Maguin E van de Guchte M Extensive horizontal transfer of core genome genes between two *Lactobacillus *species found in the gastrointestinal tract BMC Evol Biol 2007 7 141 10.1186/1471-2148-7-141 17708761 PMC1994166

[B185] Cai H Thompson R Budinich MF Broadbent JR Steele JL Genome sequence and comparative genome analysis of *Lactobacillus casei*: insights into their niche-associated evolution Genome Biol Evol 2009 1 239 257 20333194 10.1093/gbe/evp019 PMC2817414

[B186] Douillard FP Kant R Ritari J Paulin L Palva A de Vos WM Comparative genome analysis of *Lactobacillus casei *strains isolated from Actimel and Yakult products reveals marked similarities and points to a common origin Microb Biotechnol 2013 6 576 587 10.1111/1751-7915.12062 23815335 PMC3918159

[B187] Linares DM Geertsma ER Poolman B Evolved *Lactococcus lactis *strains for enhanced expression of recombinant membrane proteins J Mol Biol 2010 401 45 55 10.1016/j.jmb.2010.06.002 20542040

[B188] Bachmann H Starrenburg MJ Molenaar D Kleerebezem M van Hylckama Vlieg JET Microbial domestication signatures of *Lactococcus lactis *can be reproduced by experimental evolution Genome Res 2012 22 115 124 10.1101/gr.121285.111 22080491 PMC3246198

[B189] Bachmann H Fischlechner M Rabbers I Barfa N Branco dos Santos F Molenaar D Teusink B Availability of public goods shapes the evolution of competing metabolic strategies Proc Natl Acad Sci USA 2013 110 14302 14307 10.1073/pnas.1308523110 23940318 PMC3761572

[B190] Kleerebezem M Hols P Bernard E Rolain T Zhou M Siezen RJ Bron PA The extracellular biology of the lactobacilli FEMS Microbiol Rev 2010 34 199 230 10.1111/j.1574-6976.2009.00208.x 20088967

[B191] Brown J de Vos WM DiStefano PS Dore J Huttenhower C Knight R Lawley TD Raes J Turnbaugh P Translating the human microbiome Nat Biotechnol 2013 31 304 308 10.1038/nbt.2543 23563424

[B192] Kuipers OP Beerthuyzen MM de Ruyter PG Luesink EJ de Vos WM Autoregulation of nisin biosynthesis in *Lactococcus lactis *by signal transduction J Biol Chem 1995 270 27299 27304 10.1074/jbc.270.45.27299 7592991

[B193] Barrangou R Fremaux C Deveau H Richards M Boyaval P Moineau S Romero DA Horvath P CRISPR provides acquired resistance against viruses in prokaryotes Science 2007 315 1709 1712 10.1126/science.1138140 17379808

[B194] Desguin B Goffin P Viaene E Kleerebezem M Martin-Diaconescu V Maroney MJ Declercq J-P Soumillion P Hols P Lactate racemase is a nickel-dependent enzyme activated by a widespread maturation system Nat Commun 2014 5 10.1038/ncomms4615 PMC4066177 24710389

[B195] Kant R Paulin L Alatalo E de Vos WM Palva A Genome sequence of *Lactobacillus amylovorus *GRL1112 J Bacteriol 2011 193 789 790 10.1128/JB.01365-10 21131492 PMC3021218

[B196] Mazé A Boël G Zúñiga M Bourand A Loux V Yebra MJ Monedero V Correia K Jacques N Beaufils S Complete genome sequence of the probiotic *Lactobacillus casei *strain BL23 J Bacteriol 2010 192 2647 2648 10.1128/JB.00076-10 20348264 PMC2863562

[B197] Morita H Toh H Fukuda S Horikawa H Oshima K Suzuki T Murakami M Hisamatsu S Kato Y Takizawa T Comparative genome analysis of *Lactobacillus reuteri *and *Lactobacillus fermentum *reveal a genomic island for reuterin and cobalamin production DNA Res 2008 15 151 161 10.1093/dnares/dsn009 18487258 PMC2650639

[B198] Pridmore RD Berger B Desiere F Vilanova D Barretto C Pittet AC Zwahlen MC Rouvet M Altermann E Barrangou R The genome sequence of the probiotic intestinal bacterium *Lactobacillus johnsonii *NCC 533 Proc Natl Acad Sci USA 2004 101 2512 2517 10.1073/pnas.0307327101 14983040 PMC356981

[B199] Wang Y Wang J Ahmed Z Bai X Wang J Complete genome sequence of *Lactobacillus kefiranofaciens *ZW3 J Bacteriol 2011 193 4280 4281 10.1128/JB.05306-11 21705607 PMC3147695

[B200] Wang S Zhu H He F Luo Y Kang Z Lu C Feng L Lu X Xue Y Wang H Whole genome sequence of the probiotic strain *Lactobacillus paracasei *N1115, isolated from traditional chinese fermented milk Genome Announc 2014 2 24625864 10.1128/genomeA.00059-14 PMC3953185

[B201] Siezen RJ Francke C Renckens B Boekhorst J Wels M Kleerebezem M van Hijum SAFT Complete resequencing and reannotation of the *Lactobacillus plantarum *WCFS1 genome J Bacteriol 2012 194 195 196 10.1128/JB.06275-11 22156394 PMC3256602

[B202] Chaillou S Champomier-Vergès MC Cornet M Crutz-Le Coq AM Dudez AM Martin V Beaufils S Darbon-Rongere E Bossy R Loux V Zagorec M The complete genome sequence of the meat-borne lactic acid bacterium *Lactobacillus sakei *23K Nat Biotechnol 2005 23 1527 1533 10.1038/nbt1160 16273110

[B203] Wegmann U O'Connell-Motherway M Zomer A Buist G Shearman C Canchaya C Ventura M Goesmann A Gasson MJ Kuipers OP Complete genome sequence of the prototype lactic acid bacterium *Lactococcus lactis *subsp. *cremoris *MG1363 J Bacteriol 2007 189 3256 3270 10.1128/JB.01768-06 17307855 PMC1855848

[B204] Paulsen IT Banerjei L Myers GS Nelson KE Seshadri R Read TD Fouts DE Eisen JA Gill SR Heidelberg JF Role of mobile DNA in the evolution of vancomycin-resistant *Enterococcus faecalis* Science 2003 299 2071 2074 10.1126/science.1080613 12663927

[B205] Qin X Galloway-Peña JR Sillanpaa J Roh JH Nallapareddy SR Chowdhury S Bourgogne A Choudhury T Muzny DM Buhay CJ Complete genome sequence of *Enterococcus faecium *strain TX16 and comparative genomic analysis of *Enterococcus faecium *genomes BMC Microbiol 2012 12 135 10.1186/1471-2180-12-135 22769602 PMC3433357

[B206] Jung JY Lee SH Jeon CO Complete genome sequence of Leuconostoc gelidum strain JB7, isolated from kimchi J Bacteriol 2012 194 6665 10.1128/JB.01806-12 23144409 PMC3497507

[B207] Jung JY Lee SH Jeon CO Complete genome sequence of Leuconostoc carnosum strain JB16, isolated from kimchi J Bacteriol 2012 194 6672 6673 10.1128/JB.01805-12 23144413 PMC3497485

[B208] Oh HM Cho YJ Kim BK Roe JH Kang SO Nahm BH Jeong G Han HU Chun J Complete genome sequence analysis of *Leuconostoc kimchii *IMSNU 11154 J Bacteriol 2010 192 3844 3845 10.1128/JB.00508-10 20494991 PMC2897357

[B209] Johansson P Paulin L Säde E Salovuori N Alatalo ER Björkroth KJ Auvinen P Genome sequence of a food spoilage lactic acid bacterium, Leuconostoc gasicomitatum LMG 18811T, in association with specific spoilage reactions Appl Environ Microbiol 2011 77 4344 4351 10.1128/AEM.00102-11 21571876 PMC3127722

[B210] Dereeper A Audic S Claverie JM Blanc G BLAST-EXPLORER helps you building datasets for phylogenetic analysis BMC Evol Biol 2010 10 8 10.1186/1471-2148-10-8 20067610 PMC2821324

[B211] Dereeper A Guignon V Blanc G Audic S Buffet S Chevenet F Dufayard JF Guindon S Lefort V Lescot M Phylogeny.fr: robust phylogenetic analysis for the non-specialist Nucleic Acids Res 2008 36 W465 469 10.1093/nar/gkn180 18424797 PMC2447785

[B212] Edgar RC MUSCLE: multiple sequence alignment with high accuracy and high throughput Nucleic Acids Res 2004 32 1792 1797 10.1093/nar/gkh340 15034147 PMC390337

[B213] Castresana J Selection of conserved blocks from multiple alignments for their use in phylogenetic analysis Mol Biol Evol 2000 17 540 552 10.1093/oxfordjournals.molbev.a026334 10742046

[B214] Guindon S Gascuel O A simple, fast, and accurate algorithm to estimate large phylogenies by maximum likelihood Syst Biol 2003 52 696 704 10.1080/10635150390235520 14530136

[B215] Anisimova M Gascuel O Approximate likelihood-ratio test for branches: A fast, accurate, and powerful alternative Syst Biol 2006 55 539 552 10.1080/10635150600755453 16785212

[B216] Chevenet F Brun C Bañuls AL Jacq B Christen R TreeDyn: towards dynamic graphics and annotations for analyses of trees BMC Bioinformatics 2006 7 439 10.1186/1471-2105-7-439 17032440 PMC1615880

[B217] Charif D Lobry J Bastolla U, Porto M, Roman HE, Vendruscolo M SeqinR 1.0-2: a contributed package to the R project for statistical computing devoted to biological sequences retrieval and analysis Structural Approaches to Sequence Evolution 2007 Springer Berlin Heidelberg 207 232 Biological and Medical Physics, Biomedical Engineering

[B218] Pages H Aboyoun P Gentleman R DebRoy S Biostrings: String objects representing biological sequences, and matching algorithms 2013 R package version 2.28.0. edition

[B219] Stuben C genomes: Genome sequencing project metadata 2013 R package version 2.6.0. edition

[B220] Bik EM Eckburg PB Gill SR Nelson KE Purdom EA Francois F Perez-Perez G Blaser MJ Relman DA Molecular analysis of the bacterial microbiota in the human stomach Proc Natl Acad Sci USA 2006 103 732 737 10.1073/pnas.0506655103 16407106 PMC1334644

[B221] Booijink CCGM Zoetendal EG Kleerebezem M de Vos WM Microbial communities in the human small intestine: coupling diversity to metagenomics Future Microbiol 2007 2 285 295 10.2217/17460913.2.3.285 17661703

